# TXA_2_ attenuates allergic lung inflammation through regulation of Th2, Th9, and Treg differentiation

**DOI:** 10.1172/JCI165689

**Published:** 2024-03-14

**Authors:** Hong Li, J. Alyce Bradbury, Matthew L. Edin, Artiom Gruzdev, Huiling Li, Joan P. Graves, Laura M. DeGraff, Fred B. Lih, Chiguang Feng, Erin R. Wolf, Carl D. Bortner, Stephanie J. London, Matthew A. Sparks, Thomas M. Coffman, Darryl C. Zeldin

**Affiliations:** 1 Division of Intramural Research, National Institute of Environmental Health Sciences/NIH, Research Triangle Park, North Carolina, USA.; 2 Department of Nephrology, Duke University Medical Center, Durham, North Carolina, USA.; 3 Program in Cardiovascular and Metabolic Disorders, Duke-NUS Medical School, Singapore.

**Keywords:** Immunology, Pulmonology, Allergy, Eicosanoids, T cells

## Abstract

In lung, thromboxane A_2_ (TXA_2_) activates the TP receptor to induce proinflammatory and bronchoconstrictor effects. Thus, TP receptor antagonists and TXA_2_ synthase inhibitors have been tested as potential asthma therapeutics in humans. Th9 cells play key roles in asthma and regulate the lung immune response to allergens. Herein, we found that TXA_2_ reduces Th9 cell differentiation during allergic lung inflammation. Th9 cells were decreased approximately 2-fold and airway hyperresponsiveness was attenuated in lungs of allergic mice treated with TXA_2_. Naive CD4^+^ T cell differentiation to Th9 cells and IL-9 production were inhibited dose-dependently by TXA_2_ in vitro. TP receptor–deficient mice had an approximately 2-fold increase in numbers of Th9 cells in lungs in vivo after OVA exposure compared with wild-type mice. Naive CD4^+^ T cells from TP-deficient mice exhibited increased Th9 cell differentiation and IL-9 production in vitro compared with CD4^+^ T cells from wild-type mice. TXA_2_ also suppressed Th2 and enhanced Treg differentiation both in vitro and in vivo. Thus, in contrast to its acute, proinflammatory effects, TXA_2_ also has longer-lasting immunosuppressive effects that attenuate the Th9 differentiation that drives asthma progression. These findings may explain the paradoxical failure of anti-thromboxane therapies in the treatment of asthma.

## Abstract 2

Thromboxane suppresses Th9 differentiation and attenuates airway hyperresponsiveness during allergic lung inflammation in mouse models.

## Graphical abstract

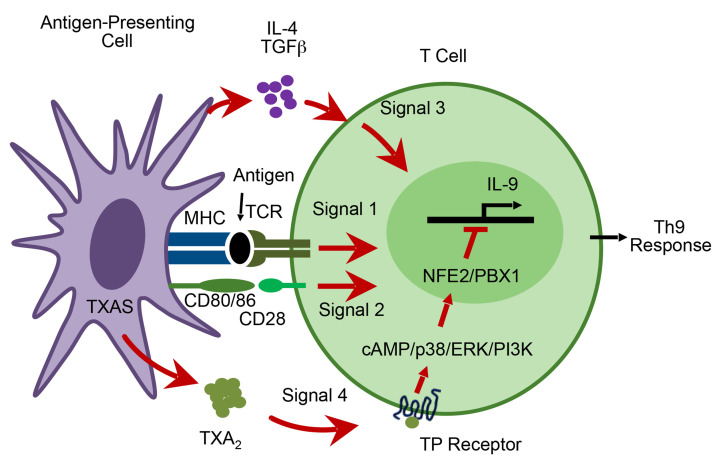

## Introduction

Development of allergic lung inflammation is a complex process involving both immune and inflammatory events. In the immune phase, allergens are taken up and processed by antigen-presenting cells such as dendritic cells (DCs), which then migrate toward regional lymph nodes. During migration, DCs become activated and undergo maturation (1, 2). Antigen-loaded mature DCs encounter naive T cells in the lymph nodes and make a physical contact referred to as an immunological synapse, through which antigen presentation and associated signaling occur (3). The strength, duration, and efficiency of this interaction determine the extent of T cell activation and differentiation (3). The interaction of T cells with DCs is known to involve 3 distinct signals (4). Signal 1 involves the interaction between major histocompatibility complex (MHC) molecules containing peptide fragments on the DC and the T cell receptor on the T cell. Signal 2 involves the interaction of costimulatory molecules (e.g., CD80/86) on the surface of the DC with ligands (e.g., CD28) on the T cell surface. Signal 3 involves the secretion of cytokines by the DC that drive T cell differentiation to unique T cell subsets.

A T helper subset called Th9 cells can differentiate either from naive T cells or from Th2 cells in the presence of both TGF-β and IL-4. TGF-β and IL-4 act through the PU.1 and interferon regulatory factor 4 (IRF4) transcription factors to induce Th9 cells to produce IL-9 and IL-10 (5, 6). IL-9 plays a pivotal role in the pathogenesis of asthma by promoting eosinophil activation and enhancing IgG/IgE production by B cells. Like Th2 cells, Th9 cells also produce IL-5 and IL-13, which induce airway hyperresponsiveness (AHR) (7–9). Interestingly, anti–IL-9 blocking antibodies inhibit allergic airway inflammation and hyperresponsiveness in mouse models and have been examined in clinical trials for treatment of humans with asthma (10, 11).

We previously demonstrated that several cyclooxygenase-2–derived (COX-2–derived) prostaglandins (PGs), including PGD_2_, PGE_2_, PGF_2α_, and PGI_2_, regulate Th17 and Th9 cell differentiation in the allergic lung (12, 13). Thromboxane A_2_ (TXA_2_) is produced by the sequential actions of cyclooxygenase-1 (COX-1) or COX-2 and thromboxane synthase (TXAS; encoded by the *TBXAS1* gene) (14). TXA_2_ is chemically unstable, with a biological half-life of approximately 30 seconds. TXA_2_ was initially identified in platelets and has potent prothrombotic and vasoconstrictive properties; as such it has been mainly studied in the cardiovascular system (15, 16). While TXAS is most abundant in platelets, it is also highly expressed in other bone marrow–derived immune cells including mast cells, granulocytes, monocytes, and macrophages (17). TXAS expression in both monocytes and macrophages results in TXA_2_ production upon cell activation (18, 19). TXA_2_ is elevated in bronchoalveolar lavage fluid (BALF) from allergic lungs (20); however, the role of TXA_2_ in Th cell differentiation and function during allergic lung inflammation remains unknown.

Like other arachidonic acid–derived signaling molecules, TXA_2_ exerts its actions through a specific G protein–coupled receptor termed the thromboxane receptor (TP receptor, encoded by the *TBXA2R* gene) (21). TXA_2_ and its receptor are present in many cell types, including cortical epithelial cells and DCs in the thymus (22, 23). Among immune cells, the TP receptor is predominantly expressed in immature CD4^–^CD8^–^ and CD4^+^CD8^+^ thymocytes and naive CD4^+^ T cells (24, 25). In the lung, the TP receptor is expressed in bronchial airway smooth muscle cells and other cell types (26). TXA_2_ induces expression of adhesion molecules in vascular endothelial cells and of eotaxin-1 (CCL-11) by bronchial smooth muscle and stimulates monocyte formation of TNF-α, IL-1β, IL-2, IL-5, and IFN-γ (27, 28). TXA_2_ induces bronchoconstriction, mucin secretion, plasma extravasation, vascular smooth muscle constriction, and vascular smooth muscle proliferation and exacerbates AHR (15, 16, 29). As a result, TP receptor antagonists and TXAS inhibitors have been developed as potential asthma therapeutics in humans (30, 31).

In this study, we used TP receptor agonists and antagonists and TP receptor–deficient (TP^–/–^) mice to investigate the role of TXA_2_ in Th9 cell differentiation and function in vitro and during allergic lung inflammation in vivo. We found that DCs express TXAS and produce TXA_2_ that signals through the TP receptor on differentiating T cells. To further elucidate the signaling cascade through which TXA_2_ regulates Th9 cell differentiation, we examined cAMP and MAPK signaling pathways and interrogated the *Il9* promoter using luciferase assays and transcription factor–specific ChIP analyses. Our results show that TXA_2_/TP receptor signaling suppresses Th9 cell differentiation through recruitment of the NFE2 and PBX1 transcriptional repressors to the *Il9* promoter. Thus, while TXA_2_ is well known for inducing inflammation and bronchoconstriction, our studies reveal what we believe to be a novel immunosuppressive role of this eicosanoid. These results help explain the failure of anti-thromboxane therapies and suggest that targeting the TXA_2_/TP receptor signaling pathway may lead to the development of novel asthma treatments.

## Results

### TXA_2_ attenuates Th9 cell responses to allergen exposure in vivo.

**Figure 1:**
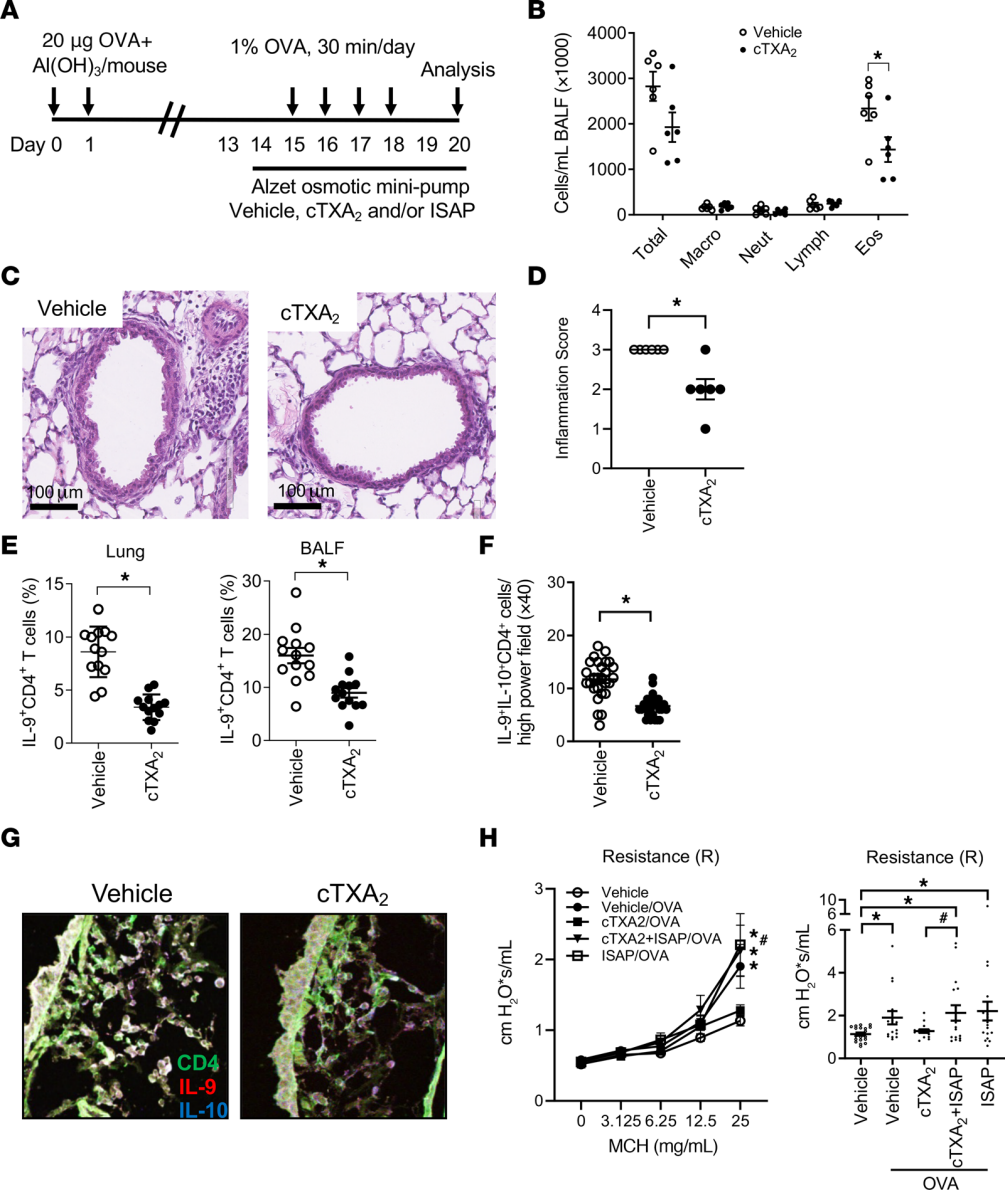
cTXA_2_ attenuates Th9 cell responses to allergen exposure in vivo. (**A**) Mice were sensitized with OVA/alum and exposed to OVA in the presence of vehicle or cTXA_2_ and/or ISAP (delivered by osmotic minipumps) as indicated. (**B**) Total cell number and cell differentials in BALF were analyzed 48 hours after the last airway OVA exposure. *n* = 6 per group, *P < 0.05. (**C**) H&E-stained lung sections from vehicle- and cTXA_2_-treated mice after OVA sensitization/exposure. Scale bars: 100 μm. Images are shown at original magnification ×40. (**D**) Scoring of lung sections revealed decreased inflammation in lungs from cTXA_2_-treated mice compared with vehicle-treated controls. *n* = 6 per group, *P < 0.05. Note that the lack of an error bar in the vehicle group is because all vehicle-treated lungs received a score of 3, i.e., 30%–50% of the lung involved in inflammation. (**E**) IL-9^+^CD4^+^ T cells, as a percentage of CD4^+^ cells in the lung and BALF after OVA-induced allergic lung inflammation. *n* = 12–13 mice per group, *P < 0.05. (**F** and **G**) Th9 cells in mouse lung tissue sections were visualized by immunofluorescence staining using anti–IL-9, anti–IL-10, and anti-CD4 antibodies. Quantitation of the number of IL-9^+^IL-10^+^CD4^+^ T cells per high-power field (HPF). (**F**) and representative ×40 images (**G**). *n* = 7 lungs per group, 5 HPFs per lung, *P < 0.05. Images are shown at original magnification ×40. (**H**) Airway resistance (R) to increasing doses of methacholine (MCH; left) and at the 25 mg/mL MCH dose (right) of non-allergic mice (vehicle) and OVA-sensitized/exposed mice treated with either vehicle, cTXA_2_, cTXA_2_+ISAP, or ISAP alone. *n* = 15–20 per group, *P < 0.05 vs. non-allergic (vehicle), ^#^*P* < 0.05 vs. OVA-sensitized/exposed cTXA_2_-treated mice. Significance was evaluated by multiple *t* tests for **B**, *t* test for **D**–**F**, and 1-way ANOVA for **H**.

To examine the role of TXA_2_ in regulating lung Th9 cell responses during allergic lung inflammation, mice were sensitized to OVA with aluminum hydroxide (alum) adjuvant, and then exposed to OVA via the airway for 4 days in the presence of vehicle or carbocyclic TXA_2_ (cTXA_2_), a biologically stable TXA_2_ analog. Fluids and tissues were collected for analysis 48 hours after the final OVA exposure (Figure 1A). OVA sensitization and exposure induced pronounced BALF eosinophilia, which was significantly attenuated in cTXA_2_-treated mice compared with vehicle-treated controls (Figure 1B). Histological sections scored by a blinded pathologist revealed decreased inflammation in lungs from cTXA_2_-treated mice compared with vehicle-treated controls (Figure 1, C and D). IL-9^+^CD4^+^ T cells (Th9 cells) from lung, BALF, lymph nodes, blood, and spleen were quantified by FACS. Compared with mice treated with vehicle, mice implanted with cTXA_2_-containing osmotic minipumps exhibited a significant decrease in the percentage of Th9 cells in the lung following OVA exposure (Figure 1E and Supplemental Figure 1; supplemental material available online with this article; https://doi.org/10.1172/JCI165689DS1). Similar results were obtained in BALF, blood, and lymph nodes, but not spleen (Figure 1E and Supplemental Figures 1 and 2). Immunofluorescence microscopy provided independent confirmation of the FACS results. Lung tissue sections from allergic mice treated with vehicle or cTXA_2_ were stained with immunofluorescently labeled antibodies against CD4 (green), IL-9 (red), and IL-10 (blue). There were significantly fewer Th9 cells (CD4^+^IL-9^+^IL-10^+^; white overlay) in lungs of cTXA_2_-treated mice compared with vehicle-treated controls (Figure 1, F and G). While cTXA_2_ treatment reduced the overall number of Th9 cells in OVA-exposed mice, it did not reduce the mean fluorescence intensity of IL-9^+^ T cells (Supplemental Figure 3A). These data suggest that cTXA_2_ regulates Th9 cell differentiation, not IL-9 expression in differentiated Th9 cells. The density of mast cells in allergic lungs was not significantly different between vehicle- and cTXA_2_-treated mice (Supplemental Figure 4, A and B). Similarly, the number of IL-9^–^IL-10^+^CD4^+^ T cells was not significantly different between vehicle- and cTXA_2_-treated mice (Supplemental Figure 5A). Together, these results indicate that cTXA_2_ significantly reduces Th9 cell numbers during OVA-induced allergic lung inflammation in vivo.

We evaluated airway responsiveness to inhaled methacholine via flexiVent in non-allergic control mice and allergic OVA-sensitized/exposed mice treated with either vehicle, cTXA_2_, the TP receptor antagonist iodophenyl sulfonyl amino pinane TXA_2_ (ISAP), or cTXA_2_ plus ISAP by minipump (Figure 1A). Vehicle-treated OVA-sensitized/exposed mice displayed increased airway responsiveness to methacholine as determined by measurement of resistance (R) and other flexiVent parameters (Figure 1H and Supplemental Figure 6). Similarly, OVA/cTXA_2_+ISAP–treated and OVA/ISAP–treated mice also displayed AHR significantly above that of non-allergic mice. Importantly, cTXA_2_ reduced airway responsiveness to levels that were not different from those in non-OVA-sensitized/exposed controls. The reduction in AHR by cTXA_2_ was not significantly less than that in OVA/vehicle–treated mice (*P* = 0.06) but was significantly less than that in cTXA_2_- and ISAP-treated mice (*P* < 0.05). Together, these results suggest that cTXA_2_ reduces AHR during OVA-induced allergic lung inflammation in vivo through activation of the TP receptor.

Bacterial lipopolysaccharide (LPS) induces maturation of DCs and promotes T cell differentiation (32). While OVA/alum sensitization primarily induces lung eosinophilia, OVA/LPS sensitization results in lung inflammation characterized by increased numbers of macrophages, neutrophils, and lymphocytes (33). Compared with vehicle, cTXA_2_ treatment significantly reduced BALF neutrophil numbers but did not reduce overall lung inflammation following sensitization with OVA/LPS and airway exposure to OVA (Supplemental Figure 7, A and B). However, significant reductions in Th9 cells were observed in lungs of cTXA_2_-treated mice relative to vehicle-treated controls (Supplemental Figure 7C). cTXA_2_ treatment also significantly reduced Th9 cells in BALF, blood, and lymph nodes, but not spleen (Supplemental Figure 7C). Thus, cTXA_2_ suppressed Th9 cells in both the OVA/alum and the OVA/LPS models of allergic lung inflammation in vivo.

### TP-deficient mice have increased lung Th9 cells after allergen exposure.

**Figure 2:**
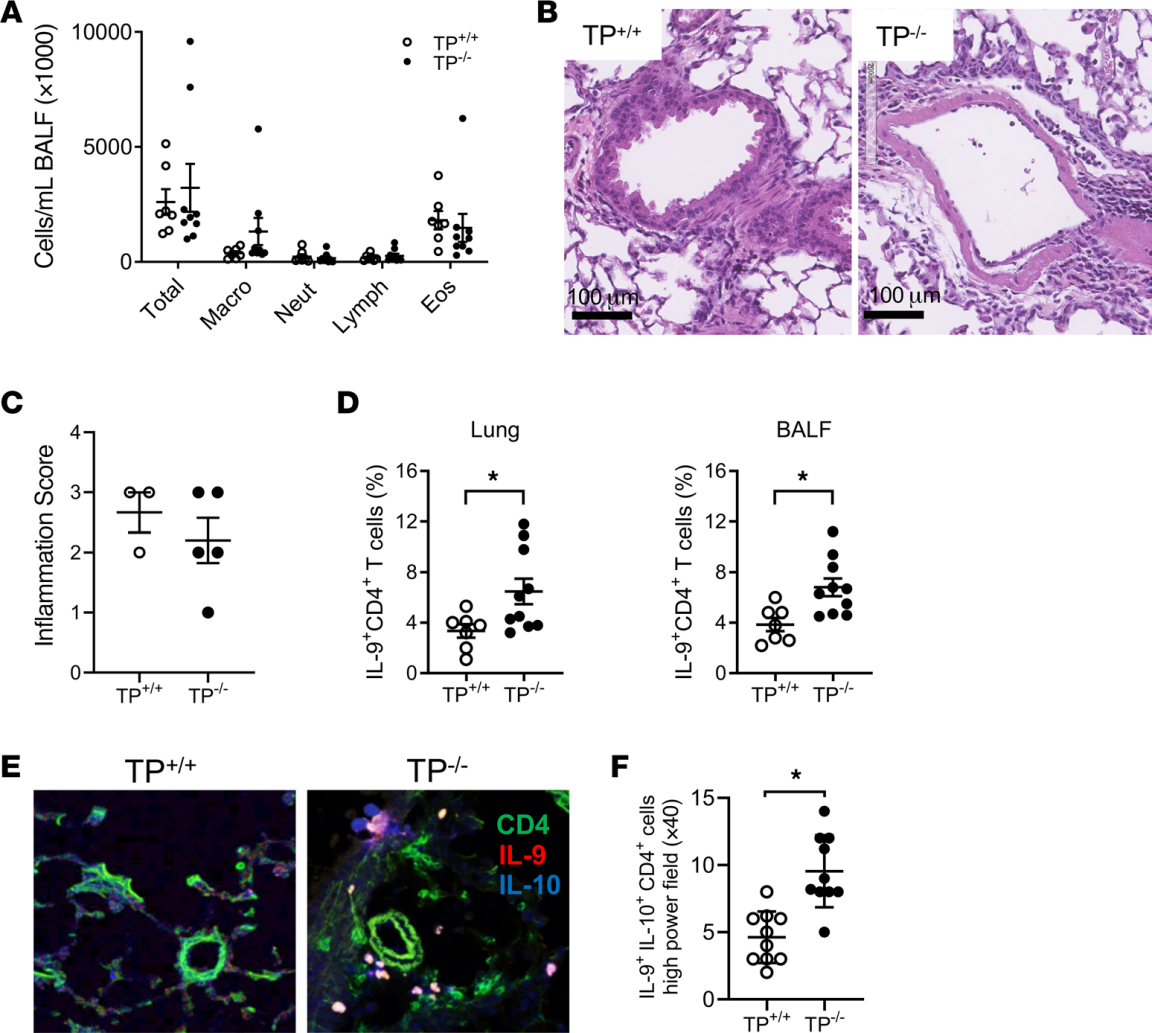
TP^–/–^ mice have increased Th9 cell responses to allergen exposure in vivo. TP^+/+^ and TP^–/–^ mice were sensitized to OVA/alum and exposed to OVA via the airway as described in Methods. (**A**) Total cell number and cell differentials from BALF were analyzed 48 hours after the last OVA exposure. *n* = 10 per group. (**B**) H&E-stained lung sections from TP^+/+^ and TP^–/–^ mice after OVA sensitization/exposure. Scale bars: 100 μm. (**C**) Scoring of lung sections revealed no differences in overall inflammation between allergic TP^+/+^ and TP^–/–^ lungs. *n* = 8 per group. (**D**) Percentage IL-9^+^CD4^+^ T cells in the lung and BALF after OVA-induced allergic lung inflammation. *n* = 7–10 mice per group, *P < 0.05. (**E**) Th9 cells in mouse lung tissue sections were visualized by immunofluorescence staining using anti–IL-9, anti–IL-10, and anti-CD4 antibodies. Images are shown at original magnification ×40. (**F**) Quantitation of the number of IL-9^+^IL-10^+^CD4^+^ T cells in lung. *n* = 5 lungs per group, 2 HPFs per lung, *P < 0.05. Significance was evaluated by multiple *t* test for **A** and *t* test for **C**, **D**, and **F**.

T cells express the TP receptor, which is functionally coupled to distinct heterotrimeric G proteins (including Gα_q_ and G_12/13_) and participates in the activation of multiple signaling cascades (34). To confirm that endogenously produced TXA_2_ regulates Th9 cells, we compared Th9 cell numbers between TP^+/+^ and TP^–/–^ mice during OVA-induced allergic lung inflammation. After OVA/alum sensitization and airway OVA exposure, BALF total cells, macrophages, neutrophils, lymphocytes, and eosinophils were not significantly different between TP^+/+^ and TP^–/–^ mice (Figure 2A). Likewise, tissue sections showed no significant differences in overall inflammation between allergic TP^–/–^ and TP^+/+^ lungs (Figure 2, B and C). However, FACS analysis indicated that TP^–/–^ mice had significantly more Th9 cells compared with TP^+/+^ mice in lung, BALF, lymph nodes, and blood, but not in spleen (Figure 2D and Supplemental Figures 8 and 9). Consistent with these results, immunofluorescence microscopy revealed that lungs from TP^–/–^ mice had significantly more Th9 cells compared with lungs from TP^+/+^ mice (Figure 2, E and F). TP^+/+^ and TP^–/–^ mice sensitized with OVA/LPS and exposed to OVA via the airway produced similar results to those observed in the OVA/alum model; BALF cell numbers and lung inflammation were not significantly different between allergic TP^–/–^ and TP^+/+^ lungs (Supplemental Figure 10, A and B). However, lungs, BALF, and lymph nodes from allergic TP^–/–^ mice had significantly more Th9 cells than corresponding tissues from allergic TP^+/+^ mice (Supplemental Figure 10C). TP disruption did not alter the mean fluorescence intensity of lung or BALF IL-9^+^ cells compared with wild type (Supplemental Figure 3B). Neither mast cell numbers nor IL-9^–^IL-10^+^CD4^+^ T cell numbers were significantly different between lungs from allergic TP^–/–^ versus TP^+/+^ mice (Supplemental Figure 4C and Supplemental Figure 5B). Together, these results suggest that endogenous TXA_2_ signaling through its canonical TP receptor attenuates Th9 cell numbers in both the OVA/alum and the OVA/LPS models of allergic lung inflammation in vivo.

### TXA_2_ inhibits Th9 cell differentiation in vitro.

**Figure 3:**
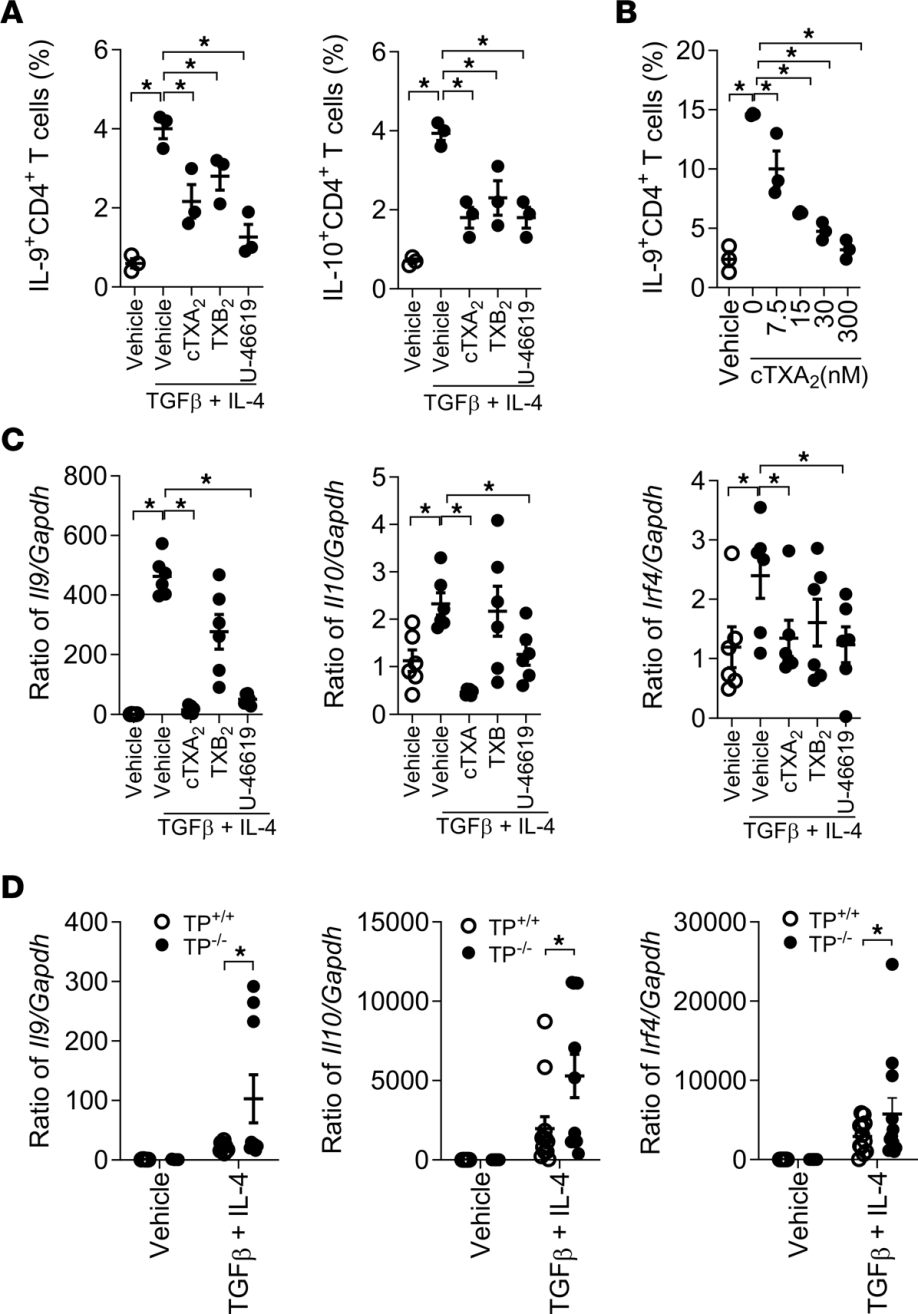
TXA_2_ inhibits Th9 cell differentiation in vitro. (**A**) Naive CD4^+^CD62L^+^ T cells were purified by FACS and treated with TGF-β and IL-4 in the presence of anti-CD28 and anti-CD3 to induce Th9 cell differentiation in vitro. During differentiation, cells were treated with either vehicle, 300 nM cTXA_2_, 300 nM U-46619 (TP receptor agonist), or 500 nM TXB_2_ (stable TXA_2_ metabolite). Compared with vehicle-treated controls, treatment with cTXA_2_, TXB_2_, or U-46619 significantly attenuated differentiation to IL-9^+^CD4^+^ and IL-10^+^CD4^+^ T cells. *n* = 3 per group, *P < 0.05. (**B**) Inhibition of Th9 differentiation by cTXA_2_ was dose dependent with significant effects at 7.5 nM. *n* = 3 per group, *P < 0.05. (**C**) cTXA_2_ and U-46619 also decreased *Il9*, *Il10*, and *Irf4* mRNA levels during Th9 differentiation. *n* = 6 per group, *P < 0.05. (**D**) Naive CD4^+^CD62L^+^ T cells from either TP^+/+^ or TP^–/–^ mice were treated with vehicle or TGF-β and IL-4 to induce Th9 cell differentiation. Th9 cell differentiation was enhanced in TP^–/–^ T cells compared with TP^+/+^ T cells as demonstrated by increased *Il9*, *Il10*, and *Irf4* mRNA levels. *n* = 5–6 per group, *P < 0.05. Significance was evaluated by 1-way ANOVA for **A**–**C** and *t* test for **D**.

**Figure 4:**
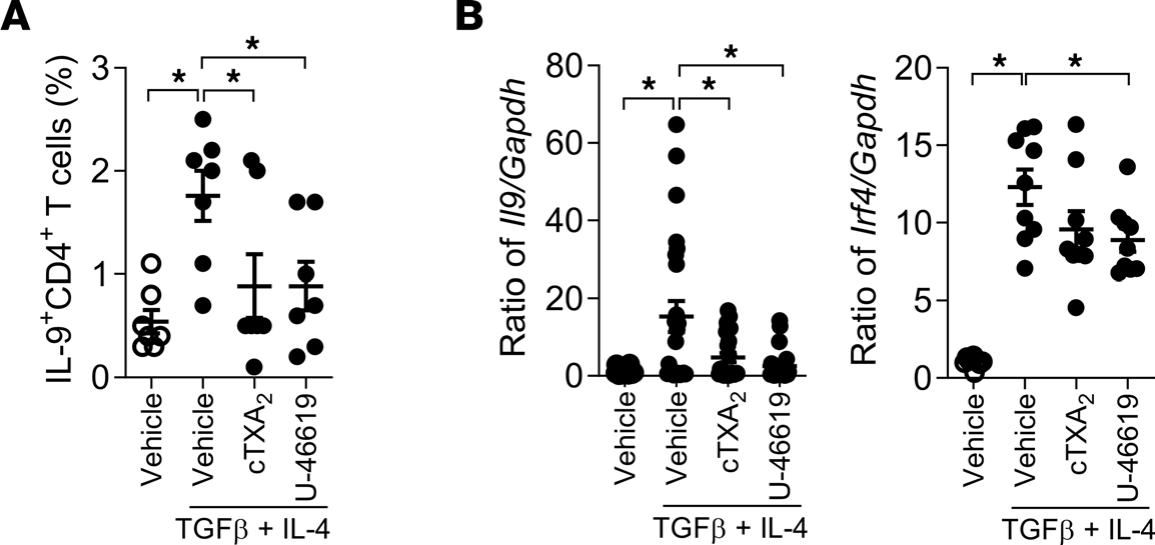
TXA_2_ inhibits human Th9 cell differentiation in vitro. Naive CD4^+^ T cells were purified from peripheral blood of healthy subjects and differentiated to Th9 cells with TGF-β and IL-4. cTXA_2_ and U-46619 significantly attenuated human Th9 cell differentiation as determined by percentages of IL-9^+^CD4^+^ cells by FACS analysis (**A**) and *Il9*, *Il10*, and *Irf4* mRNA levels (**B**). *n* = 5–12 per group, *P < 0.05. Significance was evaluated by 1-way ANOVA.

To examine the role of TXA_2_ in Th9 cell differentiation in vitro, we isolated naive CD4^+^CD62L^+^ T cells by fluorescence-activated cell sorting (FACS, >99% pure) from mouse spleens and induced them to the Th9 cell phenotype using TGF-β and IL-4 in the presence of vehicle, 300 nM cTXA_2_, 500 nM TXB_2_ (stable TXA_2_ metabolite), or 300 nM U-46619 (TP receptor agonist). As shown in Figure 3A, only 0.6% ± 0.1% of untreated naive T cells differentiated to Th9 cells after 5 days in culture. Treatment with TGF-β and IL-4 induced 4.0% ± 0.3% of naive T cells to differentiate into Th9 cells. Interestingly, cTXA_2_, TXB_2_, and U-46619 significantly inhibited Th9 cell differentiation (Figure 3A and Supplemental Figure 11). cTXA_2_ suppressed Th9 cell differentiation in a dose-dependent manner with significant effects at concentrations as low as 7.5 nM, which are physiologically relevant (Figure 3B). Consistent with these findings, *Il9*, *Il10*, and *Irf4* mRNA levels were significantly reduced by cTXA_2_ and U-46619 (Figure 3C). Moreover, TP^–/–^ naive T cells exhibited significantly increased Th9 cell differentiation compared with TP^+/+^ naive T cells as evidenced by increased *Il9*, *Il10*, and *Irf4* mRNA levels (Figure 3D). Importantly, both cTXA_2_ and U-46619 also inhibited Th9 cell differentiation of naive T cells isolated from peripheral blood of healthy human volunteers as determined by both FACS (Figure 4A) and mRNA analyses (Figure 4B and Supplemental Figure 12). Therefore, in both mouse and human, differentiation of naive T cells to Th9 cells was significantly attenuated by cTXA_2_ and a TP receptor agonist in vitro.

### TXA_2_ suppresses DC-mediated promotion of Th9 cell differentiation in vitro.

**Figure 5:**
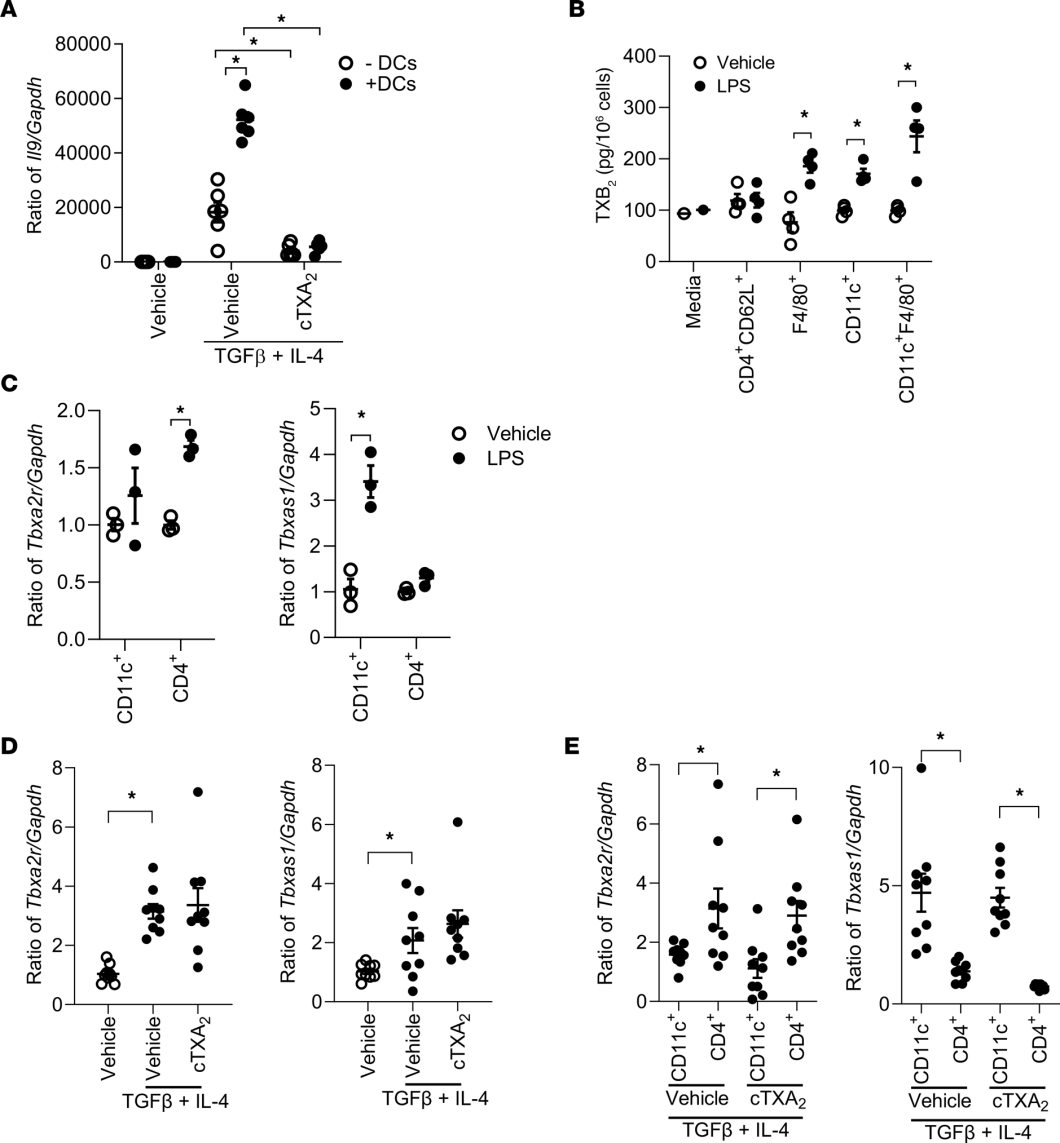
TXA_2_ inhibits promotion of Th9 cell differentiation by DCs in vitro. (**A**) Coculture of purified naive CD4^+^CD62L^+^ T cells with CD11c^+^ DCs (from lung) enhanced Th9 cell differentiation compared with naive T cells alone. Treatment with 300 nM cTXA_2_ significantly impaired Th9 cell differentiation of naive T cells, whether cultured alone or in the presence of DCs. *n* = 10 per group, *P < 0.05. (**B**) Purified naive CD4^+^CD62L^+^ T cells or CD11c^+^, CD11c^+^F4/80^+^, and F4/80^+^ myeloid cells were treated with vehicle or LPS (1 mg/mL) in vitro, and supernatants were assayed for TXB_2_ by liquid chromatography–tandem mass spectrometry. (**C**) Purified CD11c^+^ or naive CD4^+^ T cells were treated with vehicle or LPS (1 mg/mL) in vitro. LPS treatment increased TP receptor (*Tbxa2r*) mRNA levels in CD4^+^ T cells and TXA_2_ synthase (*Tbxas1*) mRNA levels in CD11c^+^ cells. *n* = 3 per group, *P < 0.05. (**D** and **E**) Mixed cultures of CD11c^+^ and CD4^+^ T cells (**D**) or Transwell-separated CD11c^+^ and CD4^+^ T cells (**E**) were incubated with vehicle, cTXA_2_, and TGF-β plus IL-4 as indicated and assayed for *Tbxa2r* and *Tbxas1* mRNA levels. *n* = 9, *P < 0.05. Significance was evaluated by 1-way ANOVA for **A** and **D** and multiple *t* tests for **B**, **C**, and **E**.

DCs are central to the orchestration of various forms of immunity. DCs modulate T cell differentiation through cytokine signaling and membrane receptors (35). DCs can mature into functionally different effector cells that potentiate Th cell subset differentiation through the specific cytokines they secrete (36–41). TXA_2_ is known to suppress low-avidity interactions between DCs and T cells and reduce DC-mediated T cell migration and proliferation (42, 43); however, it remains unknown whether TXA_2_ also inhibits DC-mediated promotion of Th9 cell differentiation from naive T cells. To address this question, we differentiated FACS-purified, naive CD4^+^CD62L^+^ T cells with or without CD11c^+^F4/80^–^ DCs in the presence or absence of cTXA_2_. Similar to the data in Figure 3, differentiation of isolated naive T cells to Th9 cells was inhibited by cTXA_2_ (Figure 5A, open circles). Coculture of DCs with naive T cells significantly potentiated Th9 cell differentiation, and this differentiation was still suppressed by cTXA_2_ (Figure 5A, filled circles).

### Myeloid cells secrete TXA_2_ and Th9 cells express the TP receptor.

Specific cell signature markers can identify myeloid cell subsets in murine lungs, including alveolar macrophages, interstitial macrophages, monocytes, and DCs (44). We isolated different lung myeloid cell subsets using CD11c and F4/80 markers and also isolated naive CD4^+^CD62L^+^ T cells by FACS. None of the cell types produced significant amount of TXB_2_ (the stable TXA_2_ metabolite) under unstimulated conditions (Figure 5B, open circles). After activating them with LPS for 24 hours, we observed that only monocytes/interstitial macrophages (F4/80^+^), DCs (CD11c^+^), and alveolar macrophages (CD11c^+^F4/80^+^) produced significant amounts of TXB_2_, but not naive T cells (Figure 5B, filled circles). Consistent with the pattern of TXB_2_ formation, LPS induced thromboxane synthase (*Tbxas1*) in CD11c^+^ cells but not naive T cells (Figure 5C). Conversely, LPS induced the TP receptor (*Tbxa2r*) in naive T cells but not CD11c^+^ cells, similar to a previous report (43). Similar responses were observed in CD11c^+^ DCs and naive T cells treated with TGF-β and IL-4. Treatment of isolated naive T cells with TGF-β and IL-4 increased *Tbxa2r* mRNA levels and suppressed *Tbxas1* mRNA levels (Supplemental Figure 13). In mixed cultures, TGF-β and IL-4 induced expression of both *Tbxa2r* and *Tbxas1* mRNAs, but cTXA_2_ treatment did not significantly alter the expression of either gene (Figure 5D). In cells cocultured with TGF-β and IL-4 but separated using Transwells, CD4^+^ T cells expressed higher levels of *Tbxa2r* than CD11c^+^ cells, while CD11c^+^ cells expressed higher levels of *Tbxas1* than CD4^+^ cells (Figure 5E). Taken together, these data suggest that CD11c^+^ DCs can be stimulated to produce TXA_2_, which activates the TP receptor on CD4^+^ T cells to modulate differentiation.

### TXA_2_ regulates both Th2 and Th9 cell differentiation.

**Figure 6:**
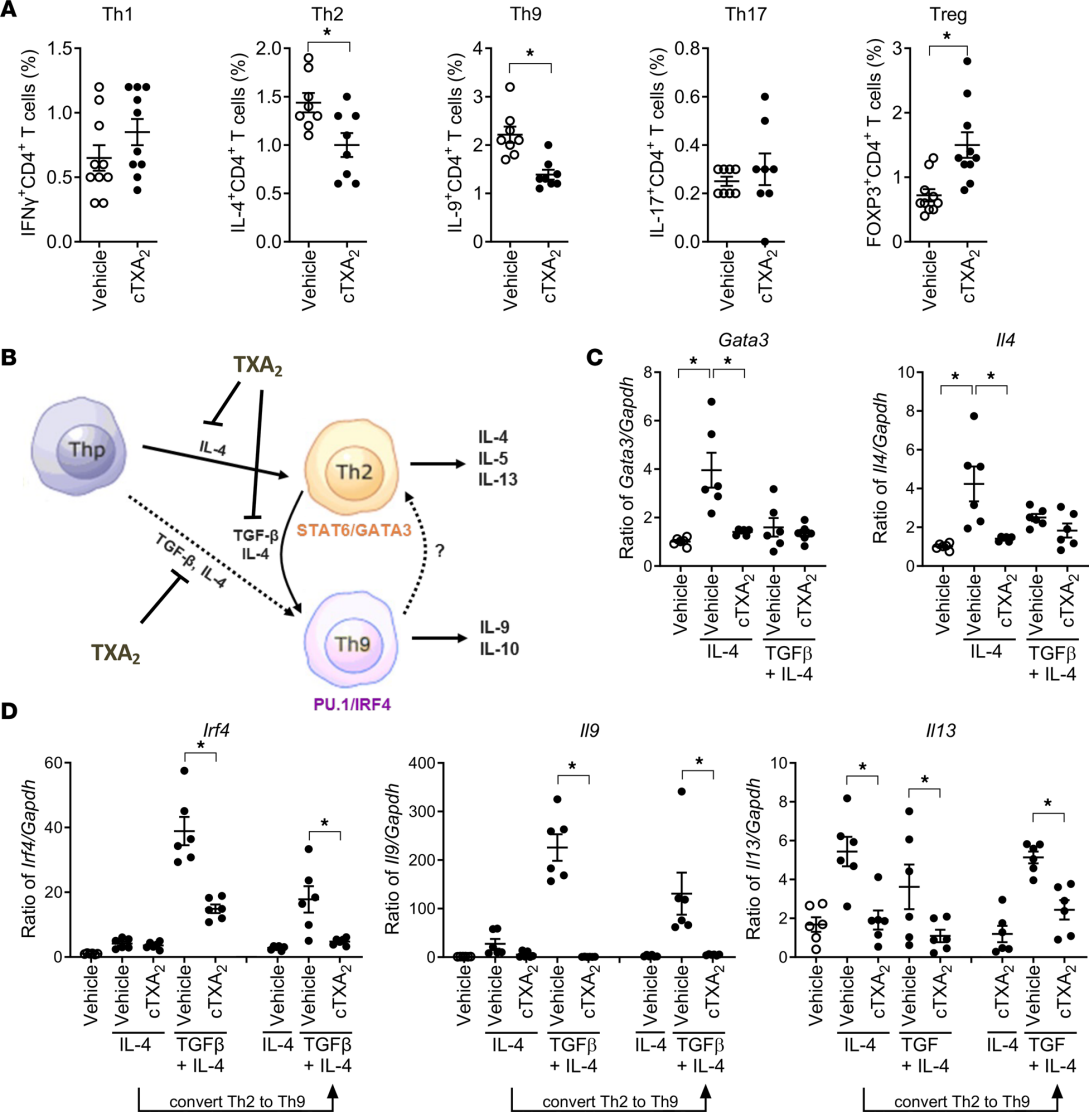
TXA_2_ inhibits Th2 cell differentiation in vivo and in vitro. Mice were sensitized with OVA/alum and exposed to OVA via the airway as depicted in Figure 1A. (**A**) Forty-eight hours after the last OVA exposure, the percentages of Th1, Th2, Th9, Th17, and Treg cells were determined by FACS analysis. *n* = 8, *P < 0.05. (**B**) Th9 cells can differentiate directly from naive T cells or from Th2 cells. (**C**) IL-4 alone, but not TGF-β and IL-4, induced Th2 cell differentiation from naive T cells as determined by mRNA levels of Th2 markers *Gata3* and *Il4*. cTXA_2_ treatment attenuated Th2 cell differentiation from naive T cells. (**D**) TGF-β and IL-4, but not IL-4 alone, induced Th9 cell differentiation from naive T cells as determined by mRNA levels of Th9 markers *Irf4*, *Il9*, and *Il13*. cTXA_2_ attenuated Th9 cell differentiation directly from naive T cells. Naive T cells were treated with IL-4 alone to generate Th2 cells, and then the Th2 cells were treated with TGF-β and IL-4 to differentiate them to Th9 cells. cTXA_2_ treatment attenuated differentiation of Th2 cells to Th9 cells. *n* = 5, *P < 0.05. Significance was determined by *t* tests for **A** and 1-way ANOVA for **C** and **D**.

To determine whether TXA_2_ regulates other Th cell subsets in vivo, mice were sensitized with OVA/alum and then exposed to OVA via the airway in the presence of vehicle- or cTXA_2_-loaded minipumps as depicted in Figure 1A. Forty-eight hours after the last OVA exposure, lungs were collected, and the percentages of CD4^+^IFN-γ^+^ (Th1), CD4^+^IL-4^+^ (Th2), CD4^+^IL-9^+^ (Th9), CD4^+^IL-17^+^ (Th17), and CD4^+^FOXP3^+^ (Treg) cells were determined by FACS. Lungs from cTXA_2_-treated mice had a significantly reduced percentage of Th2 and Th9 cells compared with lungs from vehicle-treated mice (Figure 6A). In contrast, the Th1 and Th17 cell percentages were not affected by cTXA_2_. Interestingly, lungs from cTXA_2_-treated mice had a significantly increased percentage of Tregs compared with lungs from vehicle-treated mice (Figure 6A). The cTXA_2_-induced reduction of Th2 and Th9 cells in allergic mice was reversed by the TP receptor antagonist ISAP (Supplemental Figure 14). cTXA_2_ also attenuated the increase in BALF levels of the Th2 cytokine IL-4 in vivo in allergic mice (Supplemental Figure 15).

Naive T cells can differentiate to Th9 cells via 2 pathways (45); they can directly differentiate to Th9 cells in the presence of IL-4 and TGF-β, or they can differentiate to Th9 cells in a 2-step process that involves differentiation of naive T cells to Th2 cells in the presence of IL-4, and then differentiation of Th2 cells to Th9 cells with the addition of TGF-β (Figure 6B). TXA_2_ may regulate Th9 differentiation at either step of the process, or it may regulate all 3 steps. To determine whether TXA_2_ can regulate Th2 cell differentiation, we incubated naive T cells (MACS, >95% pure) with IL-4 alone in the presence or absence of cTXA_2_. IL-4 induced the canonical Th2 transcription factor *Gata3* and the Th2 cytokine *Il4* (Figure 6C). Expression of *Gata3*, *Il4*, and *Il13* was significantly inhibited by cTXA_2_, suggesting that cTXA_2_ regulates Th2 cell differentiation from naive T cells (Figure 6, C and D). To determine whether TXA_2_ can regulate Th9 cell differentiation, we incubated naive T cells with IL-4 and TGF-β together in the presence or absence of cTXA_2_. Incubation of naive T cells with TGF-β and IL-4 induced expression of the Th9 cell markers *Irf4* and *Il9* (Figure 6D) with minimal induction of the Th2 cell markers *Gata3* and *Il4* (Figure 6C). cTXA_2_ attenuated differentiation of naive T cells directly to Th9 cells. Finally, to determine whether TXA_2_ also can inhibit differentiation of Th2 cells to Th9 cells, we first differentiated naive T cells to Th2 cells using IL-4, and then treated the Th2 cells with IL-4 and TGF-β in the presence or absence of cTXA_2_. Incubation of naive T cells with IL-4 initially, then IL-4 and TGF-β together, induced expression of the Th9 cell markers *Irf4* and *Il9* (Figure 6D). cTXA_2_ attenuated differentiation of Th2 cells to Th9 cells. Therefore, cTXA_2_ regulates differentiation of naive T cells to Th2 cells, naive T cells to Th9 cells, and Th2 cells to Th9 cells. Th9 cell differentiation is also dependent on the transcription factors BATF and STAT6 (46, 47). Interestingly, cTXA_2_ suppressed *Batf* and *Stat6* induction only after naive T cells were differentiated toward the Th2 phenotype with IL-4 or after Th2 to Th9 cell differentiation (Supplemental Figure 16). *Batf* and *Stat6* were both induced during Th9 cell differentiation by TGF-β plus IL-4; however, this was not a point of regulation by cTXA_2_.

**Figure 7:**
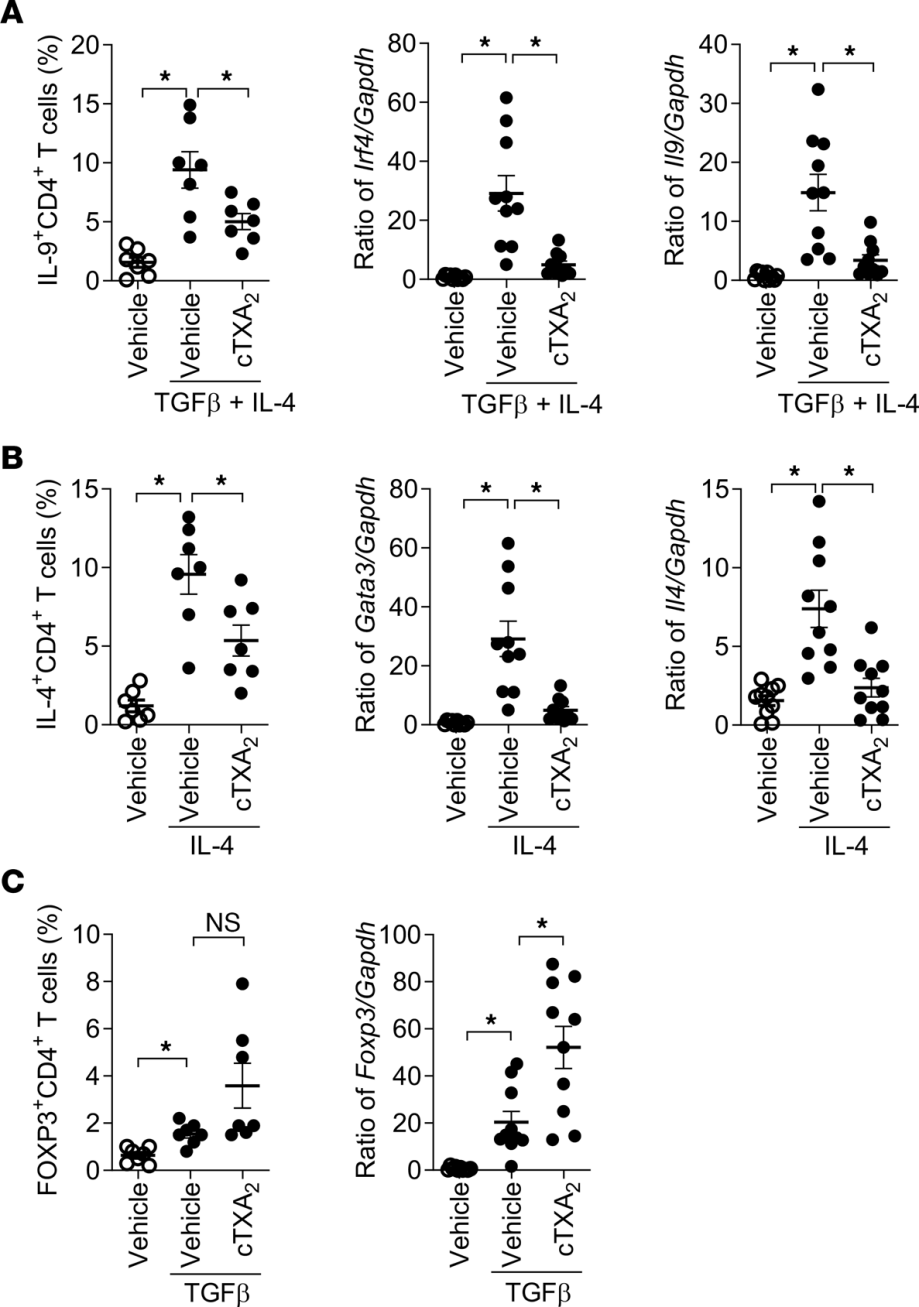
cTXA_2_ decreases human Th2 and Th9 cell differentiation and increases Treg cell differentiation in vitro. Naive CD4^+^ T cells were purified from peripheral blood of healthy subjects and differentiated to Th2 cells with IL-4, to Th9 cells with TGF-β plus IL-4, or to Tregs with TGF-β. (**A**) cTXA_2_ significantly attenuated human Th9 differentiation as determined by percentages of IL-9^+^CD4^+^ cells and expression of *Irf4* and *Il9* mRNA. (**B**) cTXA_2_ significantly attenuated human Th2 cell differentiation as determined by percentages of IL-4^+^CD4^+^ cells and expression of *Gata3* and *Il4* mRNAs. (**C**) cTXA_2_ treatment during TGF-β–induced Treg differentiation revealed a non-significant increase in FOXP3^+^CD4^+^ cells, and a similar but significant increase in *Foxp3* mRNA levels. *n* = 8 per group, *P < 0.05. Significance was determined by 1-way ANOVA for **A**–**C**.

Thromboxane also regulates differentiation of naive human CD4^+^ T cells to multiple T helper subsets. Naive T cells isolated from human peripheral blood were differentiated to Th2, Th9, and Treg cell subsets in vitro and analyzed by FACS and mRNA analyses. In an independent replication of the human T cell responses shown in Figure 4, IL-4 and TGF-β increased Th9 cells (IL-4^+^CD4^+^) and induced *Irf4* and *Il9* mRNAs, effects that were significantly attenuated by cTXA_2_ treatment (Figure 7A). Similarly, IL-4 increased Th2 cells (IL-4^+^CD4^+^) and induced *Gata3* and *Il4* mRNAs, effects that were also significantly attenuated by cTXA_2_ (Figure 7B). TGF-β increased Tregs (FOXP3^+^CD4^+^) and induced *Foxp3* mRNA (Figure 7C). Interestingly, cTXA_2_ treatment resulted in a non-significant increase in Tregs and caused a further significant induction of *Foxp3* mRNA (Figure 7C). These data suggest that cTXA_2_ regulates differentiation of naive T cells to Th2, Th9, and Treg cell subsets in both mice and humans.

### Involvement of cAMP/PKA and p38 signaling cascades in Th9 differentiation.

**Figure 8:**
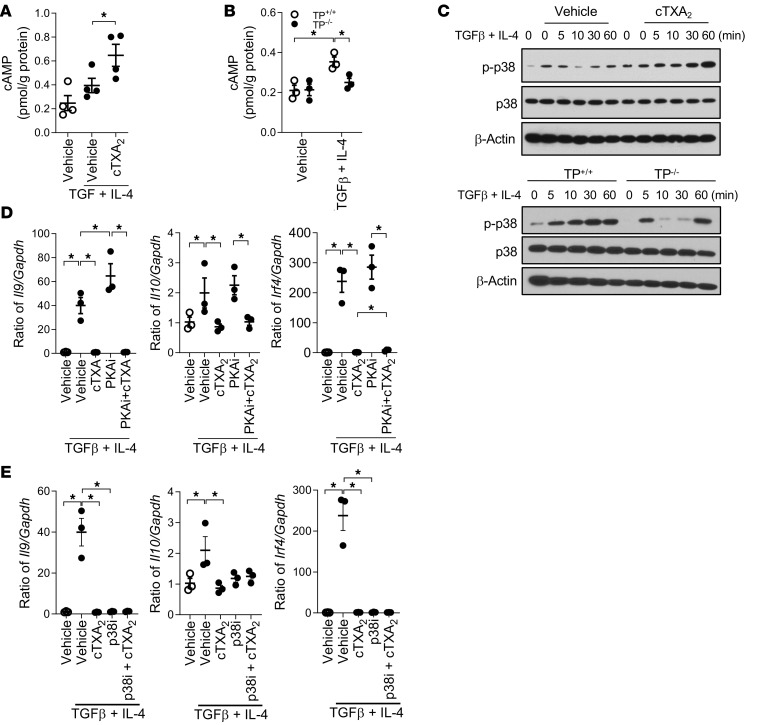
Role of cAMP/PKA and p38 MAPK signaling in Th9 cell differentiation. (**A**) Naive T cells were incubated with TGF-β and IL-4 to induce Th9 cell differentiation in the presence or absence of cTXA_2_, and intracellular cAMP levels were measured by ELISA. cAMP levels were increased by cTXA_2_ during Th9 cell differentiation. *n* = 5 per group, *P < 0.05. (**B**) Naive T cells from TP^+/+^ and TP^–/–^ mice were incubated with vehicle or TGF-β and IL-4 to induce Th9 cell differentiation. cAMP levels were increased during Th9 cell differentiation in TP^+/+^ cells (open circles), but not in TP^–/–^ cells (filled circles). *n* = 5 per group, *P < 0.05. (**C**) Treatment with cTXA_2_ enhanced phosphorylation of p38 MAPK in naive T cells incubated with TGF-β and IL-4. Incubation of naive T cells from TP^+/+^ mice with TGF-β and IL-4 increased phosphorylation of p38 MAPK (representative of 3 experiments), an effect that was attenuated in naive T cells isolated from TP^–/–^ mice (single experiment). (**D**) Naive T cells were incubated with TGF-β and IL-4 in the presence or absence of cTXA_2_ and a PKA inhibitor (PKAi). Although Th9 cell differentiation (as measured by expression of *Il9*, *Il10*, and *Irf4* expression) was modestly enhanced by PKAi, it did not significantly alter the ability of cTXA_2_ to inhibit Th9 cell differentiation. *n* = 5, *P < 0.05. (**E**) Naive T cells were incubated with TGF-β and IL-4 in the presence or absence of cTXA_2_ and a p38 MAPK inhibitor (SB203580, p38i). p38i abolished Th9 cell differentiation (as measured by expression of *Il9*, *Il10*, and *Irf4* expression). *n* = 5, *P < 0.05. Significance was determined by 1-way ANOVA for **A**–**E**.

TXA_2_ can signal through TP receptors to activate a variety of signaling cascades, including cAMP/PKA (cAMP-dependent protein kinase) and p38 MAPK pathways (48), which can interact with a variety of transcription factors, including NFE2 and PBX1, to regulate cell differentiation (49). To identify the downstream TXA_2_/TP receptor signaling pathways involved in Th9 cell differentiation, we first examined the effect of cTXA_2_ treatment and TP receptor knockout on intracellular cAMP levels in vitro. As shown in Figure 8A, cTXA_2_ increased cAMP levels by approximately 50% compared with vehicle in naive T cells treated with TGF-β and IL-4 to induce Th9 cell differentiation. Moreover, treatment of TP^+/+^ naive T cells with TGF-β and IL-4 resulted in increased intracellular cAMP levels; however, this increase did not occur when TP^–/–^ naive T cells were treated with TGF-β and IL-4 (Figure 8B). Next, we examined the effect of cTXA_2_ on phosphorylation of p38 MAPK. cTXA_2_ enhanced p38 MAPK phosphorylation in naive T cells treated with TGF-β and IL-4 (Figure 8C and Supplemental Figure 17). Consistent with these data, phosphorylation of p38 MAPK appeared reduced in TP^–/–^ naive T cells treated with TGF-β and IL-4 relative to TP^+/+^ naive T cells (Figure 8C and Supplemental Figure 17). The importance of cAMP/PKA and p38 MAPK in the cTXA_2_-mediated inhibition of Th9 cell differentiation was further examined using specific inhibitors of these signaling pathways. While protein kinase A inhibitory peptide (PKAi) modestly enhanced Th9 cell differentiation in vitro as measured by levels of *Il9*, *Il10*, and *Irf4* mRNAs, it did not significantly alter the ability of cTXA_2_ to inhibit Th9 cell differentiation (Figure 8D). We were unable to confirm the role of p38 MAPK in the cTXA_2_ effect, as the p38 MAPK inhibitor SB203580 (p38i) alone abolished Th9 cell differentiation (Figure 8E). Thus, while activation of cAMP/PKA and p38 MAPK signaling pathways may be important in Th9 cell differentiation, we cannot conclude that they are definitively involved in the TXA_2_ effect.

### TXA_2_ represses IL-9 production through PBX1 and NFE2.

**Figure 9:**
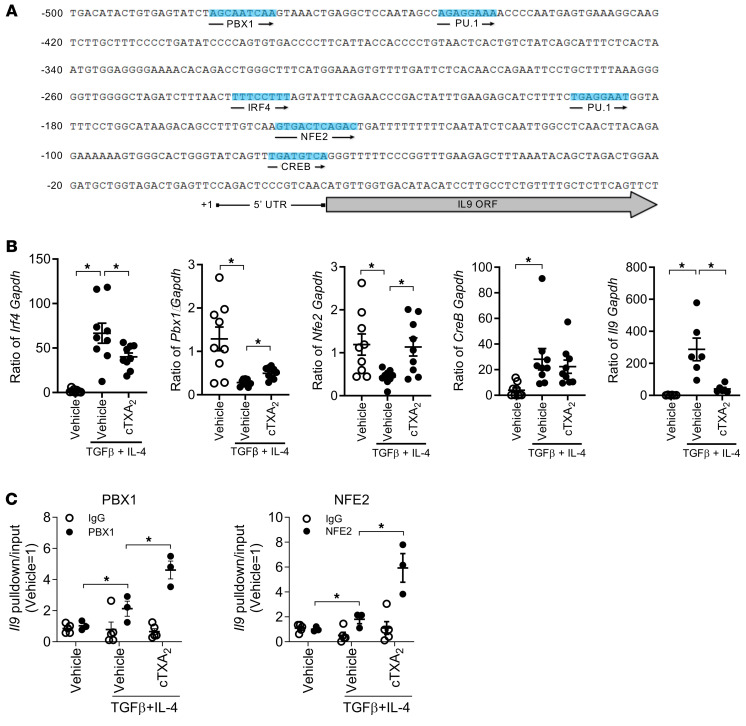
TXA_2_ alters expression/binding of PBX1 and NFE2 to the *Il9* promoter. (**A**) The locations of transcription factor binding sites in the mouse proximal *Il9* promoter. Conservation analysis and motif prediction were used to identify unique binding sites for NFE2 (–150 bp), PBX1 (–481 bp), CREB (–101 bp), PU.1 (–450 bp, –192 bp), and IRF4 (–237 bp) transcription factors relative to the transcription start site (TSS). (**B**) Naive T cells were treated with TGF-β and IL-4 to induce Th9 cell differentiation in the presence or absence of cTXA_2_, and expression of *Irf4*, *Pbx1*, *Nfe2*, *Creb*, and *Il9* mRNAs was determined by qPCR. *n* = 9, *P < 0.05. (**C**) ChIP-PCR assays of PBX1 and NFE2 binding to genomic DNA from naive T cells during Th9 cell differentiation with or without treatment with cTXA_2_. DNA fragments were pulled down with anti-NFE2, anti-PBX1, or IgG control antibodies, and NFE2- and PBX1-bound DNA was amplified using specific primers by qPCR. The percentage pull-down by NFE2 or PBX1 relative to input DNA is shown. The locations of ChIP-qPCR primers relative to the TSS (+1) and PBX1 or NFE2 binding sites are shown. *n* = 3, *P < 0.05. Significance was determined by 1-way ANOVA for **B** and multiple *t* tests for **C**.

Ultimately, Th9 cell differentiation requires upregulation of *Il9* gene transcription. The mouse *Il9* proximal promoter contains consensus binding sites for the PBX1, PU.1, IRF4, NFE2, and CREB transcription factors (Figure 9A). Prior work has shown that PU.1 and IRF4 are key transcription factors involved in Th9 cell differentiation, IL-9 production, and allergic inflammation (50). In contrast, little is known about the role of PBX1, NFE2, or CREB in regulation of Th9 cell differentiation or function. Interestingly, p38 MAPK and PKA have been reported to regulate activation and DNA binding of PBX1, NFE2, and CREB transcription factors (51–53).

We first determined the expression of these transcription factors during Th9 cell differentiation of mouse naive T cells in vitro (Figure 9B). Consistent with the role of IRF4 in Th9 cell differentiation, treatment of naive T cells with TGF-β and IL-4 increased *Irf4* mRNA expression. cTXA_2_ suppressed the induction of *Irf4* mRNA by approximately 50%. Notably, cTXA_2_ suppression of *Irf4* mRNA was less pronounced than cTXA_2_ suppression of *Il9* mRNA, suggesting that cTXA_2_ does not act solely through IRF4 to regulate Th9 cell differentiation. *Pbx1* and *Nfe2* mRNAs are both abundant in naive T cells, and treatment with TGF-β and IL-4 to induce Th9 cell differentiation decreased both *Pbx1* and *Nfe2* expression. This suggests that induction of IL-9 during Th9 cell differentiation may be through reduced expression of these known transcriptional repressors. Importantly, treatment with cTXA_2_ increased *Pbx1* expression and restored *Nfe2* expression close to that in naive T cells. Thus, suppression of IL-9 by cTXA_2_ may, at least in part, be due to restoration of the basal repression of the *Il9* promoter by PBX1 and NFE2. Treatment of naive T cells with TGF-β and IL-4 to induce Th9 cell differentiation increased expression of *Creb* mRNA; however, cTXA_2_ had no significant effect on *Creb* expression. Expression of *Pu.1* mRNA was low or undetectable in mouse naive T cells and was not changed during Th9 cell differentiation or by cTXA_2_ treatment (data not shown).

Since cTXA_2_ increased expression of *Pbx1* and *Nfe2* during Th9 cell differentiation, we examined whether it also influenced binding of these 2 transcription factors to the *Il9* promoter. We performed chromatin immunoprecipitation using PBX1- or NFE2-specific antibodies followed by quantitative PCR (qPCR) and direct sequencing of PCR products (Figure 9C and Supplemental Figure 18). cTXA_2_ significantly increased binding of both transcription factors to the immunoprecipitated chromatin. Sequencing confirmed that this binding mapped to the respective DNA sites on the *Il9* promoter.

**Figure 10:**
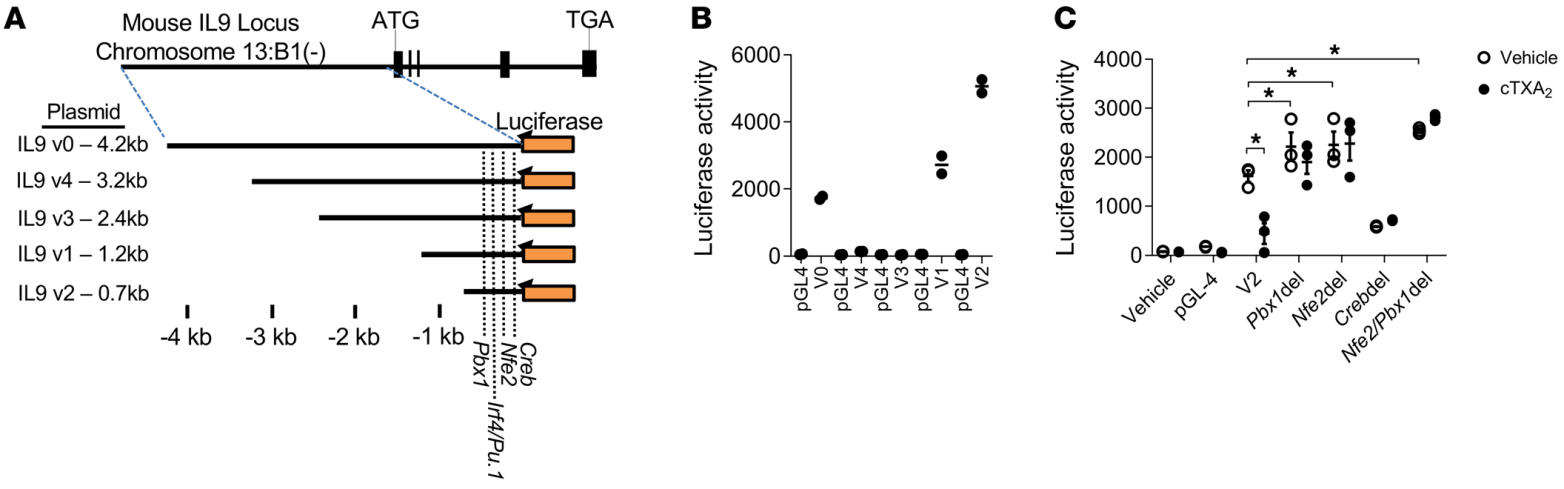
Role of PBX1 and NFE2 in *Il9* promoter activity and effect of TXA_2_. (**A**) Luciferase reporter constructs of different *Il9* promoter region fragments. Dotted lines denote locations of *Nfe2*, *Pbx1*, *Creb*, *Pu.1*, and *Irf4* response elements. (**B**) Luciferase activity of empty vector (pGL4) or truncated *Il9* promoter region fragments. The V2 construct, which contains a 700 bp promoter sequence containing consensus binding sites for the PBX1, NFE2, CREB, PU.1, and IRF4 transcription factors, showed strong luciferase activity when transfected into 293T cells. (**C**) The V2 construct was modified to delete nucleotides in the consensus *Nfe2*, *Pbx1*, and *Creb* binding sites. Compared with the parent *Il9* V2 construct, 293T cells transfected with the *Pbx1*del, *Nfe2*del, and *Nfe2/Pbx1*del constructs had increased luciferase activity, and cells transfected with the *Creb*del construct had decreased luciferase activity. Luciferase activity from the parent V2 construct was inhibited by cTXA_2_. In contrast, luciferase activity in *Pbx1*del-, *Nfe2*del-, and *Nfe2/Pbx1*del-transfected cells was not inhibited by cTXA_2_. Significance was determined by 1-way ANOVA for **B** and multiple *t* tests for **C**.

To further study the transcription factors involved in repression of *Il9* expression by TXA_2_, we made a series of luciferase reporter constructs containing varying lengths of the mouse *Il9* promoter (Figure 10A and Supplemental Table 1). All of these constructs were transfected into 293T cells, and luciferase activity was measured after correction for transfection efficiency. The V2 construct, which has a 700 bp promoter sequence containing the consensus binding sites for the PBX1, NFE2, CREB, PU.1, and IRF4 transcription factors, showed the strongest luciferase activity and was used in subsequent studies (Figure 10B). As shown in Figure 10C, the intact V2 construct had strong promoter activity, which could be significantly inhibited by cTXA_2_. This indicates that the TXA_2_-sensitive elements are contained within this V2 construct. We next used site-directed mutagenesis to disrupt the PBX1, NFE2, or CREB binding sites in the V2 promoter construct. Disruption of PBX1 or NFE2 or both binding sites increased promoter activity. This is consistent with the known repressor function of these 2 transcription factors. Importantly, disruption of NFE2 and PBX1 binding sites abolished the ability of cTXA_2_ to repress *Il9* promoter activity. Disruption of the CREB binding site reduced promoter activity, suggesting that CREB is a transcriptional activator of the *Il9* promoter. This construct was not further inhibited by cTXA_2_. Taken together, these data indicate that PBX1 and NFE2 likely mediate repression of the *Il9* promoter by TXA_2_.

## Discussion

The well-described role of TXA_2_ as a potent bronchoconstrictor suggested a novel therapeutic approach for allergic lung disease; however, TXAS inhibitors and TP receptor antagonists have shown little efficacy in the treatment of asthma patients (54). In this study, we report several findings that may help to explain this apparent paradox: (a) cTXA_2_ decreases lung inflammation, airway hyperresponsiveness, and numbers of Th2, Th9, and Treg cells in the allergic mouse lung in vivo; (b) allergic TP receptor–knockout mice have increased numbers of Th9 cells in vivo; (c) cTXA_2_ suppresses and TP receptor knockout enhances differentiation of naive T cells to Th9 cells in vitro; (d) cTXA_2_ is produced by myeloid cells and Th9 cells express the TP receptor; (e) cTXA_2_ enhances and TP receptor knockout suppresses activation of p38 MAPK and cAMP/PKA signaling pathways; and (f) cTXA_2_ induces NFE2 and PBX1 transcription factor binding to, and repression of, the *Il9* promoter. Thus, although anti-TXA_2_ therapies attenuate bronchoconstriction, they may exacerbate the asthma phenotype by promoting Th9-mediated inflammation.

Our findings add to a growing body of evidence supporting the role of TXA_2_ in regulation of immune cell function. Mouse thymus and spleen have the highest expression of TP receptors compared with other organs (55). Leung and Mihich first demonstrated that TXA_2_ had immunoregulatory; suppression of TXA_2_ production inhibited splenocyte proliferation (56). Similarly, others have shown that TXAS inhibitors and TP receptor antagonists inhibit splenocyte proliferation but lack an additive or synergistic effect (57). Both mitogen- and antigen-induced splenocyte proliferation responses are impaired in TP^–/–^ spleen cells (58). More recent studies indicated that TXA_2_/TP receptor signaling dampens acquired immunity by suppressing the interactions between DCs and T cells (43). In our studies, cTXA_2_ suppressed differentiation of purified naive T cells to Th9 cells in the presence or absence of costimulatory DCs, suggesting that it directly suppresses Th9 cell differentiation and IL-9 production, with little or no effect on DCs.

TXA_2_ appears to function in a paracrine fashion rather than an autocrine fashion to inhibit T cell differentiation. Multiple myeloid cell types are present in the lung and have been shown to regulate lung immune function (59). Our results showed that CD11c^+^ and/or F4/80^+^ myeloid cells express TXAS and produce the stable metabolite of TXA_2_ (TXB_2_) after stimulation with LPS. In contrast, the literature has conflicting evidence regarding TXAS expression and TXA_2_ production by T cells. TXAS was reported to be absent in thymic lymphocytes (24); however, TXB_2_ can be produced by some CD4^+^ T cell subsets under certain conditions (43). Our results showed that naive CD4^+^ T cells do not produce significant amounts of TXB_2_ even after LPS stimulation. In contrast, naive T cells express the TP receptor, and that expression is upregulated during Th9 differentiation. Thus, our data are consistent with a model wherein myeloid cells produce TXA_2_ that binds to the TP receptor on T cells to inhibit Th9 cell differentiation.

TXA_2_/TP receptor signaling regulates cell function through multiple signaling pathways (60, 61). The precise signaling mechanisms through which TXA_2_/TP receptor activation suppresses Th9 cell differentiation remain unknown. Our data suggest that TXA_2_/TP receptor signaling can increase cAMP levels and activate p38 MAPK during Th9 cell differentiation. Interestingly, PKA inhibition modestly enhanced Th9 cell differentiation, but it did not significantly alter the ability of cTXA_2_ to inhibit Th9 cell differentiation. Likewise, p38 MAPK inhibition alone abolished Th9 cell differentiation. Thus, while activation of cAMP/PKA and p38 MAPK may be important in Th9 cell differentiation, we cannot conclude that these signaling pathways are involved in the TXA_2_ effect.

Increased IL-9 production is a critical marker of Th9 cell differentiation; however, the network of transcription factors that mediate induction of *Il9* mRNA is incompletely understood. PU.1 (5) and IRF4 (62) transcription factors are most commonly associated with Th9 cell differentiation, although other transcription factors, including BATF (47), STAT6 (46), FOXO1 (63), ID3 (64), SIRT1 (65), and BCL6 (66), may also be involved. We examined the proximal mouse *Il9* promoter and found multiple transcription factor binding sites that could be involved in the induction of *Il9*. We focused on NFE2 and PBX1 because they were abundantly expressed in naive T cells and downregulated during Th9 cell differentiation and were known transcriptional repressors (67, 68). Interestingly, ChIP-qPCR analysis showed that cTXA_2_ induced binding of NFE2 and PBX1 to their respective sites in the *Il9* promoter. Moreover, disruption of the NFE2 and PBX1 binding motifs using site-directed mutagenesis enhanced *Il9* promoter activity and abolished the suppressive effect of cTXA_2_. Together, these data suggest that TXA_2_ inhibits *Il9* transcription, at least in part, through activation of NFE2 and PBX1.

It is noteworthy that in addition to regulating Th9 cell differentiation/function, we observed that TXA_2_ also regulates Th2 and Treg cell differentiation/function both in vitro and in vivo in both mice and humans. Th2 cells produce IL-4, IL-5, and IL-13, which are key players in the lung immune response to allergen (69). Other COX-derived eicosanoids have been shown to play important roles in modulating Th2 immunity. For example, PGE_2_ can shift the balance of CD4^+^ Th cells toward a Th2-type immune response through regulation of DCs and altering the local cytokine microenvironment (70). In contrast, others have shown that COX-2 inhibition reduces PGE_2_ formation in vivo and increases Th2-mediated lung inflammation (71). PGD_2_ has been reported to stimulate chemotaxis of Th2 cells (72). PGI_2_ analogs suppress Th2 cytokine production in an antigen-specific manner through the IP receptor (73). Together with our work, these published studies suggest that the effects of COX-derived eicosanoids on Th2 responses are complex.

The effects of TP disruption and cTXA_2_ treatment on Th9 cell differentiation and function were consistent across different in vitro and in vivo models; however, not all variables tracked well with changes in Th9 cell numbers. For example, cTXA_2_ suppressed inflammation in the OVA/alum model but not in the OVA/LPS model. Similarly, increased Th9 cells in TP-null mice did not exacerbate lung inflammation. One possible explanation for these apparent discrepancies may be that histological scoring is less sensitive and more variable than other measures of inflammation. Alternatively, it is well established that selection of the adjuvant is determinative with regard to the characteristics of the allergic response. Our data are consistent with those of others who find that LPS induces a lower level of lung inflammation than other adjuvants (33). In our experiments, OVA/alum (inflammation scores ~3; Figure 1D and Figure 2C) induced more inflammation than OVA/LPS (inflammation scores ~1; Supplemental Figure 7B and Supplemental Figure 10B). OVA/LPS also may skew allergic responses more toward Th1 or Th17 responses (74, 75). For example, in our experiments, OVA/LPS induced a neutrophilic- and lymphocytic-predominant lung inflammatory response compared with a pronounced eosinophilic response induced by OVA/alum (Supplemental Figure 7 vs. Figure 1, respectively). Our study is also limited in that it cannot elucidate whether the major suppressive effects of cTXA_2_ on airway responsiveness are due to effects on Th2, Th9, Treg, and/or other cell types such as epithelial cells. Despite these limitations, the reproducible immunomodulatory effects of cTXA_2_ across multiple in vitro and in vivo models, the consistent findings in both mice and humans, and the suppression of airway responsiveness in a clinically relevant eosinophilic-predominant allergic model give us confidence that cTXA_2_ plays an important role in the development of allergic lung inflammation.

We believe that our findings are clinically relevant since we observed similar effects of TXA_2_ in human naive T cells isolated from peripheral blood of healthy volunteers. Indeed, both cTXA_2_ and the TP receptor agonist U-46619 significantly attenuated Th9 cell differentiation of human naive T cells ex vivo, while the TP receptor antagonist ISAP was able to reverse the effects of cTXA_2_. These findings suggest the use of TP receptor agonists and/or TXAS activators for the treatment of Th9 allergic inflammation in the asthmatic lung. In addition, these observations raise the possibility that polymorphisms in the *TBXAS1* or the *TBXA2R* gene may be associated with altered asthma risk in humans. Functional polymorphisms in these 2 genes have been reported in the literature, and several published studies have examined their contribution to asthma risk. A recent meta-analysis of 7 studies concluded that the *TBXA2R 924C/T* polymorphism is associated with asthma risk and the *TBXA2R* *795C/T* polymorphism may be a risk factor for aspirin-intolerant asthma (76). Likewise, a rare allele (rs6962291) in the *TBXAS1* gene was associated with lower catalytic activity and was protective in aspirin-intolerant asthma in a Korean population (77). Further work is necessary to determine whether these or other polymorphisms in TXAS/TP receptor pathway genes are associated with allergic airway inflammation or asthma in other populations.

**Figure 11:**
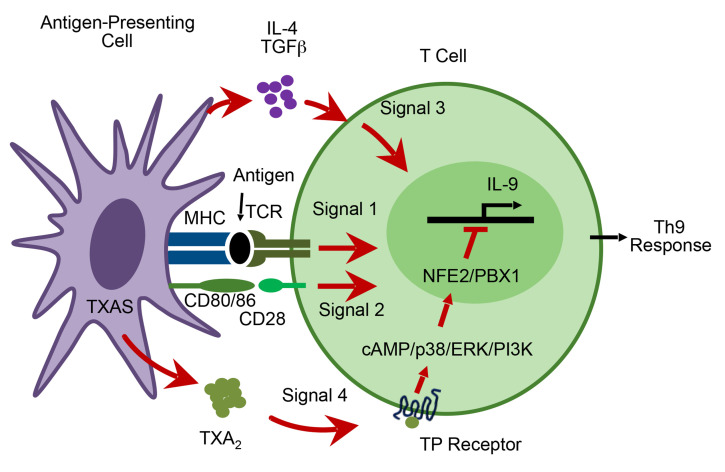
Model for TXA_2_ regulation of Th9 cell differentiation. The interaction of T cells with antigen-presenting cells involves 4 signals. Signal 1 involves the interaction between MHC molecules containing peptide fragments on the antigen-presenting cell and the T cell receptor (TCR) on the T cell. Signal 2 involves the interaction between costimulatory molecules (such as CD80/86) on the surface of the antigen-presenting cell and ligands (such as CD28) on the T cell surface. Signal 3 involves the secretion of bioactive cytokines (IL-4 and TGF-β) by the antigen-presenting cell. Signal 4 involves secretion of TXA_2_ by antigen-presenting cells that binds to the TP receptor on T cells to limit Th9 cell differentiation via a mechanism that involves the transcription factors NFE2 and PBX1.

In summary, TXA_2_/TP receptor signaling attenuates lung Th9 cell differentiation during OVA-induced allergic lung inflammation in vitro and in vivo. Within the immune synapse, TXA_2_ is produced by activated DCs and detected by differentiating T cells that express the TP receptor. We believe that this novel pathway represents a fourth signal whereby antigen-presenting cells interact with T cells to influence Th9 cell differentiation (Figure 11). Within T cells, TXA_2_ induces p38 MAPK activation, binding of NFE2 and PBX1 transcription factors to the *Il9* promoter, and suppression of *Il9* transcription. Thus, in contrast to its prothrombotic, proinflammatory, and spasmogenic effects, thromboxane exerts immunosuppressive effects that attenuate Th9 cell differentiation and IL-9 secretion during allergic lung inflammation.

## Methods

### Sex as a biological variable.

Our study exclusively examined male mice to limit variability in phenotype. Human samples were obtained from both male and female subjects. It is unknown whether the findings in mice are relevant to female mice.

### Reagents and animals.

Antibodies were purchased from BD Biosciences, eBioscience, BioLegend, and Cell Signaling Technology. Eicosanoids and inhibitors were purchased from Cayman Chemical. Other chemicals and buffers were purchased from Sigma-Aldrich. Tissue culture media and supplements were from Gibco/Thermo Fisher Scientific. Male and female C57BL/6J mice (6–10 weeks of age) were purchased from The Jackson Laboratory. Male TP^+/+^ and TP^–/–^ mice (6–10 weeks old) on a pure C57BL/6 background (backcrossed >10 generations) were provided by Thomas Coffman (Duke University).

### OVA-induced allergic airway inflammation model in vivo.

Mice were sensitized with 20 μg OVA using either 0.2 mL aluminum hydroxide (alum) or 1 μg bacterial LPS (from *Pseudomonas aeruginosa 10*, Sigma-Aldrich) as an adjuvant by intraperitoneal injection on days 0 and 1; 14–21 days later, mice were exposed to 1% OVA (≥98% pure by agarose gel electrophoresis) in saline (or saline only) via inhalation for 30 minutes per day for 4 consecutive days. Vehicle (15% ethanol in PBS), cTXA_2_, and/or iodophenyl sulfonyl amino pinane TXA_2_ (ISAP) (1 μmol/mouse/day) were delivered 1 week before airway OVA exposure via subcutaneously implanted osmotic minipumps (model 1007D, Alzet). Mice were euthanized for assessments 48 hours after the last OVA exposure.

### Lung function assessment.

Invasive lung function analysis was performed on mice with a flexiVent FX2 (SCIREQ) according to the manufacturer’s instructions as previously described (78).

### Histology and immunofluorescence staining.

Lungs were intratracheally instilled with 50% Sakura Tissue-Tek OCT compound (International Medical Equipment) at 25 cm H_2_O and frozen on dry ice. Lung sections were fixed in methanol with 0.3% H_2_O_2_ for 10 minutes at 4°C and permeabilized with Triton X-100 (0.8%) for 10 minutes at room temperature. After lung sections were blocked with 5% BSA in PBS, they were simultaneously immunostained with anti–IL-9, anti–IL-10, and anti-CD4 antibodies for 1 hour at room temperature (BioLegend, catalog 514104, 505016, and 100414). Mast cells were stained with DAPI, anti-CD45, and anti-CD117 (BioLegend 160303 and 105831). Lung sections were imaged using an Axioplan 2 fluorescence microscope (Carl Zeiss) with a digital camera (AxioCam MRC or MRM, Carl Zeiss) or a confocal microscope (LSM 710, Carl Zeiss). Lungs were scored by a blinded pathologist based on the percentage of lung involved in inflammation: 0 = no inflammation; 1 = 1%–10%; 2 = 11%–30%; 3 = 31%–50%; 4 = >50%. Cell fluorescence intensity was quantified with ImageJ software (NIH).

### In vitro differentiation of naive T cells.

Lung and spleen CD4^+^ T cells were isolated by magnetic-activated cell sorting (MACS). Naive CD4^+^ T cells were isolated using the CD4^+^CD62L^+^ isolation kit (Miltenyi Biotec; ~95% purity) or by fluorescence-activated cell sorting (FACS; ~99% purity). Human CD4^+^ T cells were isolated by MACS from peripheral blood. Cells were cultured in the presence of anti-CD3 (BioLegend 317302; 3 μg/mL) and anti-CD28 (BioLegend 302902; 1 μg/mL) and differentiated to Th1 (with IL-12, 20 ng/mL), Th2 (IL-4, 20 ng/mL), Th9 (IL-4, 20 ng/mL, and TGF-β, 2 ng/mL), Th17 (TGF-β, 10 ng/mL, and IL-6, 30 ng/mL), and Tregs (TGF-β, 10 ng/mL). In some experiments, cells were treated with cTXA_2_ (7.5–300 nM), TXB_2_ (500 nM), or TP receptor agonist U-46619 (300 nM) or corresponding vehicle.

### Flow cytometry analysis.

Single-cell suspensions from lung, spleen, and peritoneal lymph nodes were prepared by mechanical disruption. Analysis of Th9 cells was performed using anti–IL-9, anti–IL-10, and anti-CD4 antibodies (BioLegend 514104, 505016, and 100414). Anti-rat IgG was used as the negative control. Surface staining was performed by incubation of samples at 4°C for 20 minutes. 7-Aminoactinomycin D (EMD Millipore) was used to discriminate dead cells. Intracellular staining was performed with the BD Cytofix/Cytoperm Fixation/Permeabilization Solution Kit (BD Biosciences) using the PE-conjugated anti–IL-9 and APC-conjugated anti–IL-10 mAbs. Analysis of other T helper subsets was performed using anti-CD4 with IFN-γ (Th1; BioLegend 505807), IL-4 (Th2; BioLegend 50413), FOXP3 (Treg; BioLegend 126403), or IL-17 (Th17; BioLegend 506915). Samples were analyzed by FACS (BD LSRFortessa with HTS option) using LSR II and FlowJo software (Tree Star).

### Naive CD4^+^ T cell purification.

Lungs, lymph nodes, and spleens from C57BL/6J, TP^+/+^, or TP^−/−^ mice were pooled and homogenized using a 70 μm cell strainer. Red blood cells were lysed in 1 mL of a Tris-HCl pH 7.5/0.83% ammonium chloride buffer for 3 minutes. After washing with 0.2% BSA/PBS, cells were placed in a medium containing RPMI 1640 supplemented with 10% FBS, 100 U/mL penicillin, 100 U/mL streptomycin, 100 mM sodium pyruvate, l-glutamine, and nonessential amino acids. We used EasySep Mouse T Cell Negative Selection kit or Mouse CD4^+^ T Cell Enrichment kit as described in the manufacturer’s instructions (STEMCELL Technologies). Naive CD4^+^ T cells were sorted based on staining by anti–mouse CD4–APC–Cy7 (BioLegend 100414) and anti–mouse CD62L–PE (BioLegend 161204). Sorted naive CD4^+^ T cell purities were greater than 99%.

### Lung myeloid cell isolation.

Mouse lung tissue was cut into small pieces, then digested with collagenase II (0.5 mg/mL; Worthington) and DNase I (20 μg/mL; Sigma-Aldrich) at 37°C for 1 hour for preparation of single lung cells. Lung myeloid cells were enriched from the lung single-cell suspension with EasySep Mouse Pan-DC Enrichment Kit (STEMCELL Technologies). F4/80^+^ cells (monocytes/interstitial macrophages), CD11c^+^ cells (monocyte-derived DCs), and CD11c^+^/F4/80^+^ cells (alveolar macrophages) were sorted with anti–mouse F4/80–FITC (BioLegend 123108) and anti–mouse CD11c–PE (BioLegend 117307) antibodies from the enriched lung myeloid cells using a BD FACSAria II instrument.

### Eicosanoid analysis of myeloid cells and naive CD4^+^ T cells.

Purified myeloid cell subsets (CD11c^+^, F4/80^+^, and CD11c^+^F4/80^+^) and naive CD4^+^ T cells were either unstimulated or stimulated with LPS (1 mg/mL) at 37°C for 4 hours. Eicosanoid levels in the supernatants were analyzed by liquid chromatography–tandem mass spectrometry as previously described (12).

### Lung DC–T cell cocultures.

Lung DC–T cell cocultures were performed in 24-well flat-bottom culture plates. Briefly, purified DCs (CD11c^+^) from lung were treated with LPS (1 mg/mL) overnight, after which the culture supernatants were removed, and the cells were extensively washed and resuspended in RPMI/10% FCS. Naive CD4^+^ T cells (1 × 10^6^ per well) were then cocultured with the DCs (1 × 10^5^ per well) in the presence of anti-CD3 (2 μg/mL), anti-CD28 (1 μg/mL), IL-4 (20 ng/mL), and TGF-β (2 ng/mL) for 5 days in 1 mL of complete culture medium. Some experiments were performed with DCs and T cells separated in 24-well Transwell cultures (Corning Costar 3470; 0.4 μm pores).

### Measurement of intracellular cAMP concentrations.

Isolated naive T cells (2 × 10^6^/mL) from C57BL/6, TP^+/+^, and TP^–/–^ mice were treated with vehicle, cTXA_2_, and cytokines as described. cAMP levels were determined using a cAMP enzyme immunoassay kit following the manufacturer’s instructions (Cayman Chemical, catalog 581001).

### Il-9 promoter luciferase reporter assay.

The *Il9* gene transcription start site (TSS) is located on the negative strand of chromosome 13 at position 56,630,060 (mm39). Five different lengths of the proximal *Il9* promoters were PCR amplified from the C57BL/6J genome: 4.2 kb, V0 (TSS, 56,634,261); 3.2 kb, V4 (TSS, 56,633,269); 2.4 kb, V3 (TSS, 56,632,508); 1.2 kb, v1 (TSS, 56,631,308); and 0.7 kb, V2 (TSS, 56,630,769) (Supplemental Table 1). Promoter fragments were subcloned into the promoterless pGL4-10 luciferase reporter vector (Promega E6651) and transfected into Jurkat T cells (clone E6-1, ATCC TIB-152). Twenty-four hours after transfection, cells were treated with 300 nM cTXA_2_ or vehicle for 4 hours. Luciferase activity was detected using the Dual-Luciferase Reporter Assay System (Promega).

### ChIP-qPCR.

Naive CD4^+^ T cells were either untreated or treated with TGF-β and IL-4 in the presence of vehicle or 300 nM cTXA_2_. Chromatin immunoprecipitation (ChIP) assays were performed following the manufacturer’s instructions (Agarose ChIP kit 26156, Pierce/Thermo Fisher Scientific). The fragmented DNA samples obtained from the ChIP with anti-NFE2 (Abcam ab140598), anti-PBX1 (Thermo Fisher Scientific PA5-17223), and anti-IgG antibodies (Thermo Fisher Scientific PA5-31160) and input samples were amplified by qPCR with specific primers.

### qPCR.

Total RNA was isolated using the RNeasy Mini Kit (Qiagen), and cDNA was synthesized with the High-Capacity cDNA Archive Kit (Applied Biosystems). All qPCR reagents were purchased from Life Technologies. The following oligonucleotides (Applied Biosystems) were used to amplify *Il9*, *Il10*, *Irf4*, *PU.1*, and *Gapdh* TaqMan primers: *Il9*, Mm00434304_m1; *Il10*, Mm01288386_m1; *Irf4*, Mm00516431_m1; *PU.1* (*Spi1*), Mm00488428_m1; *Tbxa2r*, Mm00436917_m1; *Pbx1*, Mm04207617_m1; *Nfe2*, Mm00801891_m1; *Crtc2*, Mm01219960_m1; *Gata3*, Mm00484683_m1; *Gapdh*, Mm99999915_g1.

### PKA and p38 MAPK inhibitor studies.

Inhibitors of PKA (PKAi, Cayman Chemical; 100 nM) and p38 MAPK (SB203580, Cayman Chemical; 1 mg/mL) were used in Th9 cell differentiation studies in vitro.

### Statistics.

Data are presented as means ± SEM. Statistical comparisons among treatment groups were performed by randomized-design, 2-way ANOVA, followed by the Newman-Keuls post hoc test for more than 2 groups, or by unpaired 2-tailed Student’s *t* test for 2 groups using Prism software (GraphPad Inc.), as appropriate. Statistical significance was defined as a *P* value of less than 0.05.

### Study approval.

All animal experiments were performed according to NIH guidelines and were approved by the National Institute of Environmental Health Sciences Animal Care and Use Committee, Research Triangle Park, North Carolina, USA (95-18). Peripheral blood was collected following written informed consent under a protocol approved by the National Institute of Environmental Health Sciences Institutional Review Board, Research Triangle Park, North Carolina, USA (10-E-0063).

### Data availability.

Values for all data points in graphs are reported in the Supporting Data Values file.

## Author contributions

Hong Li designed research studies, conducted experiments, analyzed data, and wrote the manuscript. JAB and MLE performed data acquisition and data analysis and revised the manuscript. AG designed research studies and generated reagents. Huiling Li and JPG performed data acquisition and data analysis. LMD acquired data and conducted experiments. FBL, CF, ERW, CDB, and SJL performed data acquisition and data analysis. MAS acquired data and conducted experiments. TMC provided reagents. DCZ designed research studies, analyzed data, and revised the manuscript.

## Supplementary Material

Supplemental data

Unedited blot and gel images

Supporting data values

## Version 1 03/14/2024

In-Press Preview

## Version 2 05/01/2024

Electronic publication

## References

[1] Lanzavecchia A, Sallusto F (2001). Regulation of T cell immunity by dendritic cells. Cell.

[2] Na H (2016). Regulation of Th2 cell immunity by dendritic cells. Immune Netw.

[3] Grakoui A (1999). The immunological synapse: a molecular machine controlling T cell activation. Science.

[4] Curtsinger JM, Mescher MF (2010). Inflammatory cytokines as a third signal for T cell activation. Curr Opin Immunol.

[5] Gerlach K (2014). TH9 cells that express the transcription factor PU.1 drive T cell-mediated colitis via IL-9 receptor signaling in intestinal epithelial cells. Nat Immunol.

[6] Tamiya T (2013). Smad2/3 and IRF4 play a cooperative role in IL-9-producing T cell induction. J Immunol.

[7] Koch S (2017). Th9 and other IL-9-producing cells in allergic asthma. Semin Immunopathol.

[8] Shimbara A (2000). IL-9 and its receptor in allergic and nonallergic lung disease: increased expression in asthma. J Allergy Clin Immunol.

[9] Levitt RC (1999). IL-9 pathway in asthma: new therapeutic targets for allergic inflammatory disorders. J Allergy Clin Immunol.

[10] Cheng G (2002). Anti-interleukin-9 antibody treatment inhibits airway inflammation and hyperreactivity in mouse asthma model. Am J Respir Crit Care Med.

[11] Parker JM (2011). Safety profile and clinical activity of multiple subcutaneous doses of MEDI-528, a humanized anti-interleukin-9 monoclonal antibody, in two randomized phase 2a studies in subjects with asthma. BMC Pulm Med.

[12] Li H (2013). Cyclooxygenase-2 inhibits T helper cell type 9 differentiation during allergic lung inflammation via down-regulation of IL-17RB. Am J Respir Crit Care Med.

[13] Li H (2011). Cyclooxygenase-2 regulates Th17 cell differentiation during allergic lung inflammation. Am J Respir Crit Care Med.

[14] Malmsten CL (1986). Arachidonic acid metabolism in inflammation and hypersensitivity reactions: a brief introduction. Cephalalgia.

[15] Hamberg M (1975). Thromboxanes: a new group of biologically active compounds derived from prostaglandin endoperoxides. Proc Natl Acad Sci U S A.

[16] FitzGerald GA (1987). Thromboxane A2 biosynthesis in human disease. Fed Proc.

[17] Fontana P (2014). Antiplatelet therapy: targeting the TxA2 pathway. J Cardiovasc Transl Res.

[18] Tone Y (1994). Abundant expression of thromboxane synthase in rat macrophages. FEBS Lett.

[19] Brune K (1978). Pharmacological control of prostaglandin and thromboxane release from macrophages. Nature.

[20] Sayers BC (2013). Role of cyclooxygenase-2 in exacerbation of allergen-induced airway remodeling by multiwalled carbon nanotubes. Am J Respir Cell Mol Biol.

[21] Narumiya S (1999). Prostanoid receptors: structures, properties, and functions. Physiol Rev.

[22] Nusing R (1990). Immunohistochemical localization of thromboxane synthase in human tissues. Eicosanoids.

[23] Nusing R (1992). Localization of thromboxane synthase in human tissues by monoclonal antibody Tü 300. Virchows Arch A Pathol Anat Histopathol.

[24] Ushikubi F (1993). Thromboxane A2 receptor is highly expressed in mouse immature thymocytes and mediates DNA fragmentation and apoptosis. J Exp Med.

[25] Namba T (1992). Mouse thromboxane A2 receptor: cDNA cloning, expression and northern blot analysis. Biochem Biophys Res Commun.

[26] Coleman RA, Sheldrick RL (1989). Prostanoid-induced contraction of human bronchial smooth muscle is mediated by TP-receptors. Br J Pharmacol.

[27] Shi H (1998). Effect of thromboxane A2 inhibitors on allergic pulmonary inflammation in mice. Eur Respir J.

[28] Powell WS (2021). Eicosanoid receptors as therapeutic targets for asthma. Clin Sci (Lond).

[29] Devillier P, Bessard G (1997). Thromboxane A2 and related prostaglandins in airways. Fundam Clin Pharmacol.

[30] Dogne JM (2002). Therapeutic potential of thromboxane inhibitors in asthma. Expert Opin Investig Drugs.

[31] Magazine R (2018). Comparison of oral montelukast with oral ozagrel in acute asthma: a randomized, double-blind, placebo-controlled study. Lung India.

[32] McAleer JP (2007). The lipopolysaccharide adjuvant effect on T cells relies on nonoverlapping contributions from the MyD88 pathway and CD11c+ cells. J Immunol.

[33] Whitehead GS (2017). TNF is required for TLR ligand-mediated but not protease-mediated allergic airway inflammation. J Clin Invest.

[34] Bos CL (2004). Prostanoids and prostanoid receptors in signal transduction. Int J Biochem Cell Biol.

[35] Kapsenberg ML (2003). Dendritic-cell control of pathogen-driven T-cell polarization. Nat Rev Immunol.

[36] Rampal R (2016). Retinoic acid-primed human dendritic cells inhibit Th9 cells and induce Th1/Th17 cell differentiation. J Leukoc Biol.

[37] Ruocco G (2015). T helper 9 cells induced by plasmacytoid dendritic cells regulate interleukin-17 in multiple sclerosis. Clin Sci (Lond).

[38] Dejani NN (2016). Topical prostaglandin E analog restores defective dendritic cell-mediated Th17 host defense against methicillin-resistant staphylococcus aureus in the skin of diabetic mice. Diabetes.

[39] Poloso NJ (2013). PGE2 differentially regulates monocyte-derived dendritic cell cytokine responses depending on receptor usage (EP2/EP4). Mol Immunol.

[40] Myer RG (2010). Prostaglandin E2-dependent IL-23 production in aged murine dendritic cells. Exp Gerontol.

[41] Boniface K (2009). Prostaglandin E2 regulates Th17 cell differentiation and function through cyclic AMP and EP2/EP4 receptor signaling. J Exp Med.

[42] Moalli F (2014). Thromboxane A2 acts as tonic immunoregulator by preferential disruption of low-avidity CD4+ T cell-dendritic cell interactions. J Exp Med.

[43] Kabashima K (2003). Thromboxane A2 modulates interaction of dendritic cells and T cells and regulates acquired immunity. Nat Immunol.

[44] Zaynagetdinov R (2013). Identification of myeloid cell subsets in murine lungs using flow cytometry. Am J Respir Cell Mol Biol.

[45] Abdelaziz MH (2020). Th2 cells as an intermediate for the differentiation of naïve T cells into Th9 cells, associated with the Smad3/Smad4 and IRF4 pathway. Exp Ther Med.

[46] Goswami R (2012). STAT6-dependent regulation of Th9 development. J Immunol.

[47] Jabeen R (2013). Th9 cell development requires a BATF-regulated transcriptional network. J Clin Invest.

[48] Obara Y (2005). Thromboxane A2 promotes interleukin-6 biosynthesis mediated by an activation of cyclic AMP-response element-binding protein in 1321N1 human astrocytoma cells. Mol Pharmacol.

[49] Paul F (2015). Transcriptional heterogeneity and lineage commitment in myeloid progenitors. Cell.

[50] Chang HC (2010). The transcription factor PU.1 is required for the development of IL-9-producing T cells and allergic inflammation. Nat Immunol.

[51] Delghandi MP (2005). The cAMP signalling pathway activates CREB through PKA, p38 and MSK1 in NIH 3T3 cells. Cell Signal.

[52] Kilstrup-Nielsen C (2003). PBX1 nuclear export is regulated independently of PBX-MEINOX interaction by PKA phosphorylation of the PBC-B domain. EMBO J.

[53] Su YF (2012). Phosphorylation-dependent SUMOylation of the transcription factor NF-E2. PLoS One.

[54] Hernandez JM, Janssen LJ (2015). Revisiting the usefulness of thromboxane-A2 modulation in the treatment of bronchoconstriction in asthma. Can J Physiol Pharmacol.

[55] Li X, Tai HH (2013). Activation of thromboxane A2 receptor (TP) increases the expression of monocyte chemoattractant protein -1 (MCP-1)/chemokine (C-C motif) ligand 2 (CCL2) and recruits macrophages to promote invasion of lung cancer cells. PLoS One.

[56] Leung KH, Mihich E (1980). Prostaglandin modulation of development of cell-mediated immunity in culture. Nature.

[57] Davi G (2012). Thromboxane receptors antagonists and/or synthase inhibitors. Handb Exp Pharmacol.

[58] Thomas DW (2003). Proinflammatory actions of thromboxane receptors to enhance cellular immune responses. J Immunol.

[59] Kim HY (2010). The many paths to asthma: phenotype shaped by innate and adaptive immunity. Nat Immunol.

[60] Pluchart H (2017). Targeting the prostacyclin pathway: beyond pulmonary arterial hypertension. Trends Pharmacol Sci.

[61] Matsuoka T, Narumiya S (2007). Prostaglandin receptor signaling in disease. ScientificWorldJournal.

[62] Gomez-Rodriguez J (2016). Itk is required for Th9 differentiation via TCR-mediated induction of IL-2 and IRF4. Nat Commun.

[63] Buttrick TS (2018). Foxo1 promotes Th9 cell differentiation and airway allergy. Sci Rep.

[64] Nakatsukasa H (2015). The DNA-binding inhibitor Id3 regulates IL-9 production in CD4(+) T cells. Nat Immunol.

[65] Wang Y (2016). Histone deacetylase SIRT1 negatively regulates the differentiation of interleukin-9-producing CD4(+) T cells. Immunity.

[66] Bassil R (2014). BCL6 controls Th9 cell development by repressing Il9 transcription. J Immunol.

[67] Laurent A (2008). PBX proteins: much more than Hox cofactors. Int J Dev Biol.

[68] Wang Z (2021). Interplay between cofactors and transcription factors in hematopoiesis and hematological malignancies. Signal Transduct Target Ther.

[69] Webb DC (2000). Integrated signals between IL-13, IL-4, and IL-5 regulate airways hyperreactivity. J Immunol.

[70] Harizi H (2003). Prostaglandin E2 modulates dendritic cell function via EP2 and EP4 receptor subtypes. J Leukoc Biol.

[71] Laouini D (2005). COX-2 inhibition enhances the TH2 immune response to epicutaneous sensitization. J Allergy Clin Immunol.

[72] Hirai H (2001). Prostaglandin D2 selectively induces chemotaxis in T helper type 2 cells, eosinophils, and basophils via seven-transmembrane receptor CRTH2. J Exp Med.

[73] Dorris SL (2012). PGI2 as a regulator of inflammatory diseases. Mediators Inflamm.

[74] Whitehead GS (2022). A neutrophil/TGF-β axis limits the pathogenicity of allergen-specific CD4+ T cells. JCI Insight.

[75] Hammad H, Lambrecht BN (2021). The basic immunology of asthma. Cell.

[76] Pan Y (2016). Association between thromboxane A2 receptor polymorphisms and asthma risk: a meta-analysis. J Asthma.

[77] Oh SH (2011). Association analysis of thromboxane A synthase 1 gene polymorphisms with aspirin intolerance in asthmatic patients. Pharmacogenomics.

[78] House JS (2015). Genetic variation in HTR4 and lung function: GWAS follow-up in mouse. FASEB J.

