# Supplementary material for: TXA2 attenuates allergic lung inflammation through regulation of Th2, Th9, and Treg differentiation
Source: J Clin Invest. 2024 Mar 14;134(9):e165689. doi: 10.1172/JCI165689 (PMC11060738; doi:10.1172/JCI165689)
Supplement: Supplemental data [file jci-134-165689-s165.pdf]

Supplemental Figure 1

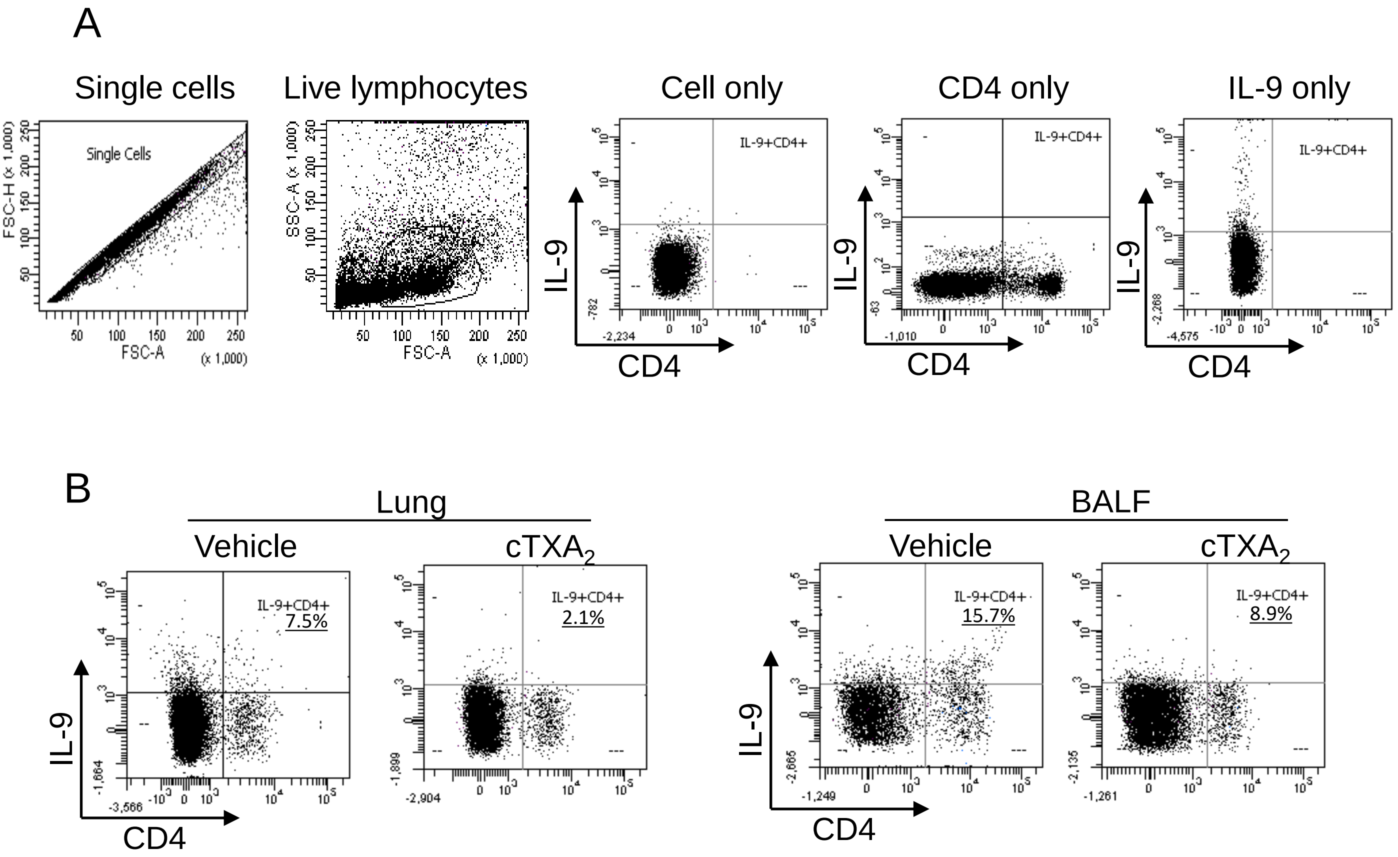

Supplemental Figure 1. A) Gating scheme for detection of IL-9$^+$CD4$^+$ T cells. Doublets and dead cells were excluded, and lymphocytes were electronically gated. IL-9+CD4+ T cells were identified as positive for both PE-conjugated anti-IL-9 and FITC-conjugated anti-CD4. B) FACs Representative FACS dot plots for the data represented in Figure 1E. Mice were sensitized with OVA/alum and exposed OVA in the presence of vehicle or cTXA$_2$ (delivered by osmotic minipumps) as indicated. IL-9$^+$CD4$^+$ T cells, as a percentage of CD4$^+$ cells in the lung and BALF after OVA-induced allergic lung inflammation (N=12-13 mice per group).

Supplemental Figure 2

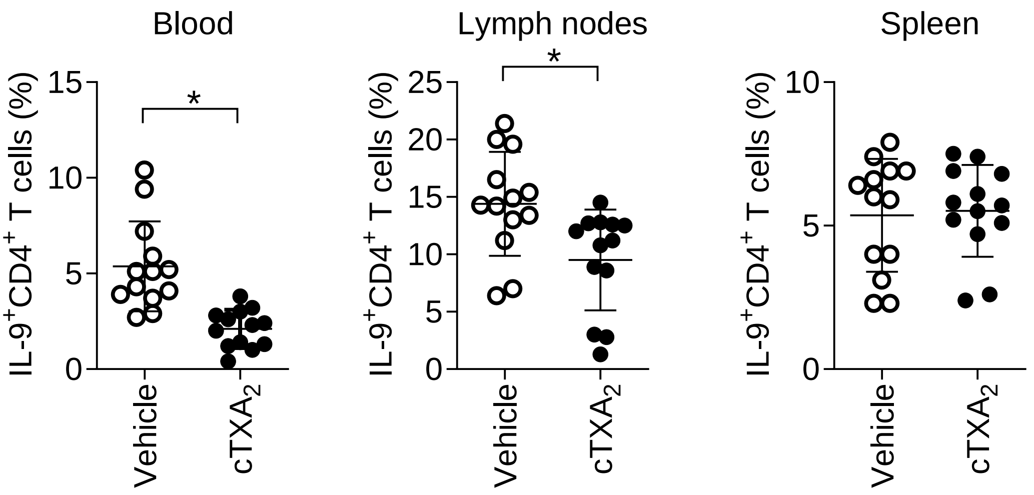

Supplemental Figure 2. $cTXA_2$ attenuates Th9 cells in blood and lymph nodes, but not spleen, during allergic lung inflammation *in vivo.* Mice were sensitized with OVA/alum and then exposed to OVA via the airway in the presence of vehicle or $cTXA_2$ (delivered by osmotic minipumps) as described in Methods. The percent of IL-9^+ $CD4^+$ T cells in different tissues was measured by flow cytometry. N=13 mice per group, *$p<0.05$.

Supplemental Figure 3

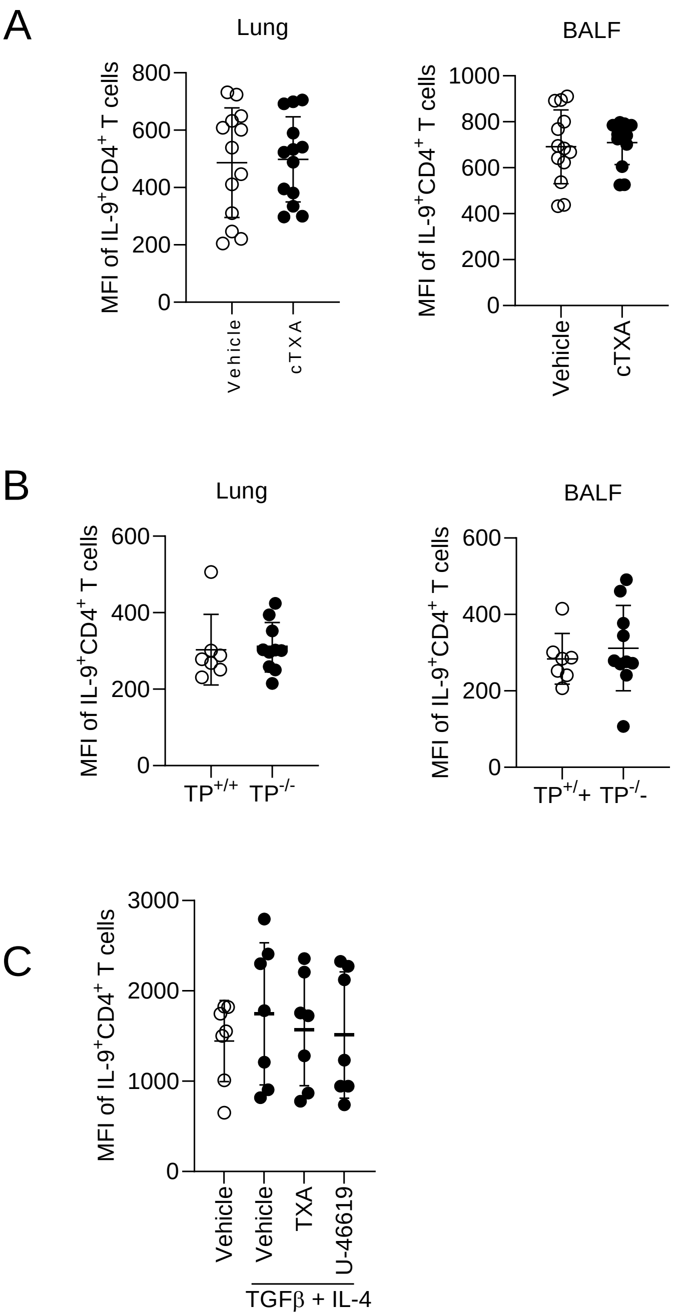

Supplemental Figure 3. Mean Fluorescence Intensity of IL-9+ CD4+ T cells. Mean Fluorescence Intensity (MFI) of IL-9+ CD4+ T cells in lung and BALF from the experiments in Figure 1E (A), Figure 2D (B) and Figure 4A (C). Each circle represents the MFI of IL-9+ CD4+ T cells from a single mouse/cell isolate as determined by flow cytometry. N=10-13 mice per group, no comparisons were statistically different.

Supplemental Figure 4

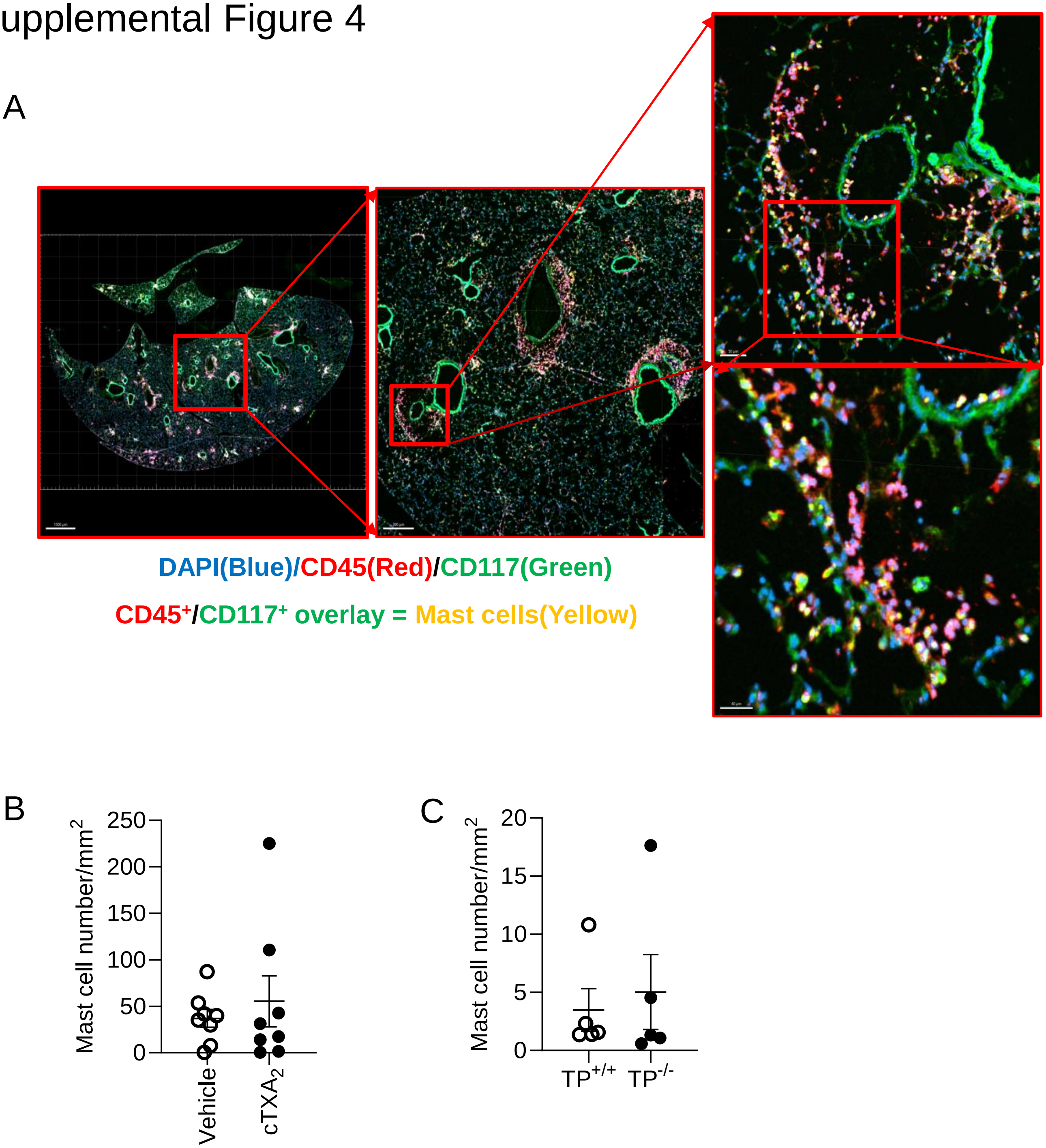

Supplemental Figure 4. Mast cell numbers in lungs of allergic mice. A) Mast cells in mouse lung tissue sections were visualized by immunofluorescent staining using DAPI, anti-CD45 (labeled with Alexa Fluor 647) and anti-CD117 (labeled with Alexa Fluor 594) antibodies. In panels B and C, each circle represents mean mast cell densities from 5 high powered fields per lung. Quantification was performed on a subset of lungs from the experiments shown in Figure 1 (B) and Figure 2 (C). N=5-7 lungs per group, no statistically significant differences were observed.

Supplemental Figure 5

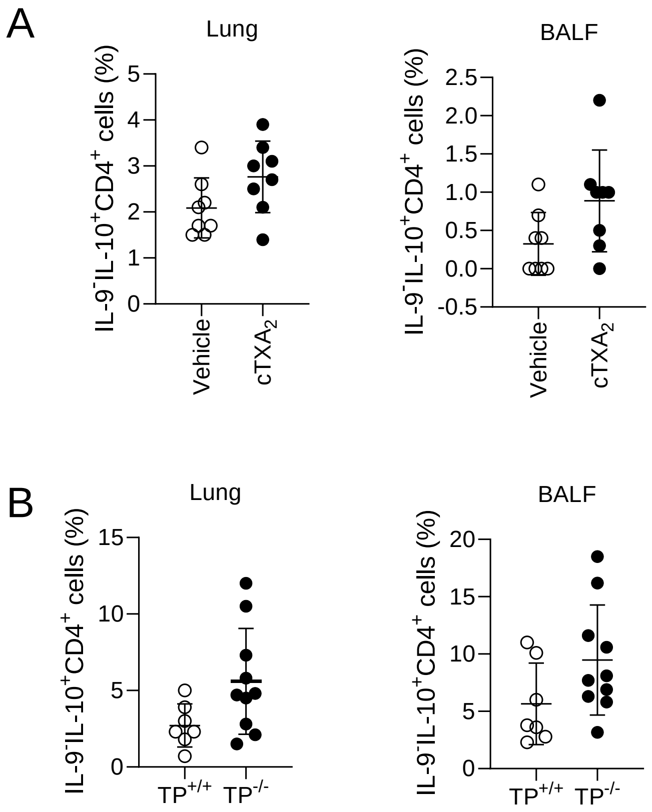

Supplemental Figure 5. Thromboxane signaling does not significantly alter the number of Th9$^-$IL10$^+$CD4$^+$ T cells in lung or BALF of allergic mice. A) Mice were sensitized/exposed to OVA and treated with either vehicle or cTXA$_2$ by minipump. B) TP$^{+/+}$ and TP$^{-/-}$ mice were sensitized/exposed to OVA. The percentage of IL-9$^-$IL10$^+$CD4$^+$ T cells in lung and BALF was determined by flow cytometry. N=7-10 mice per group, no differences were statistically significant.

Supplemental Figure 6

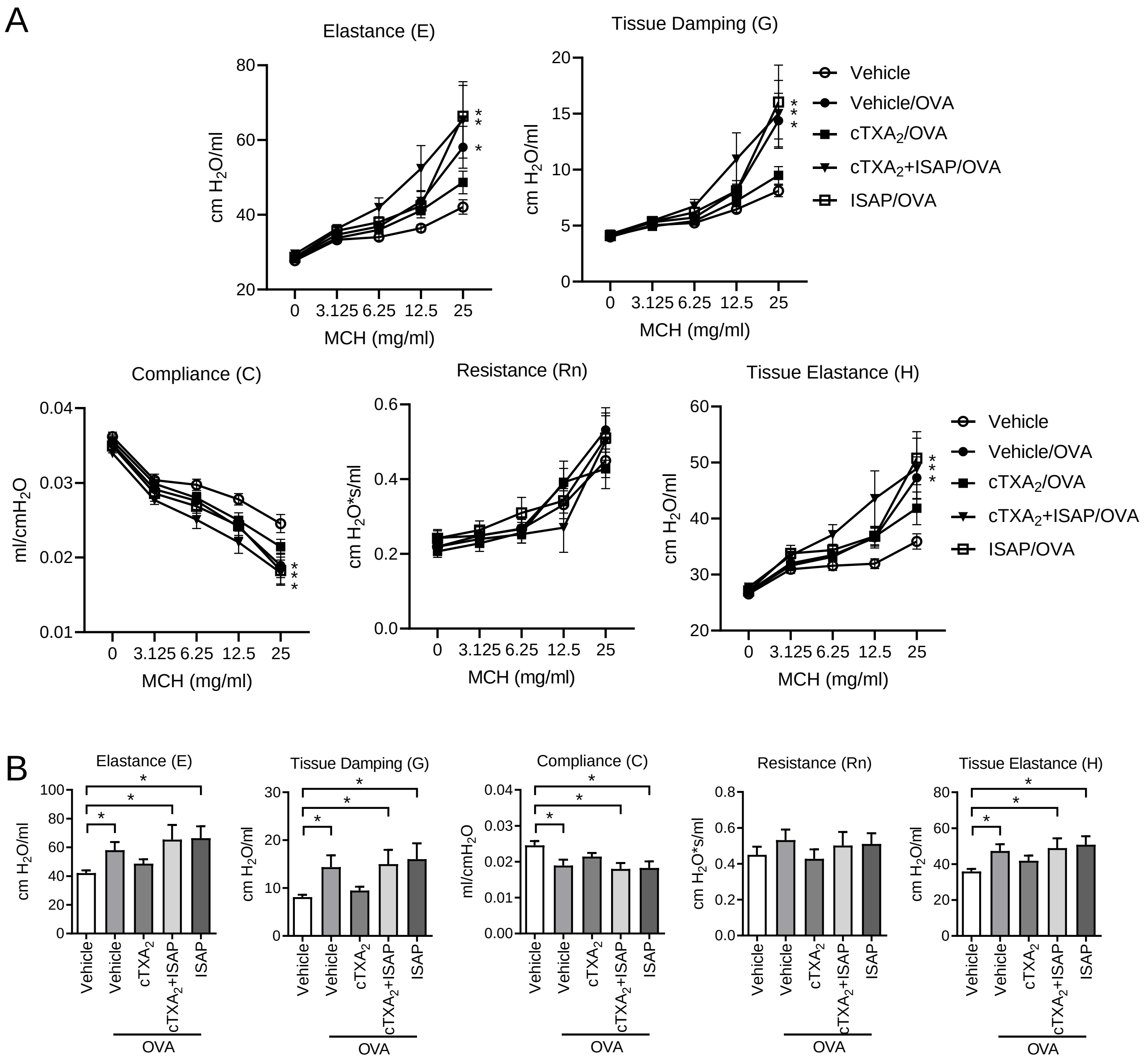

Supplemental Figure 6. Thromboxane attenuates airway responsiveness in allergic mice. WT mice were treated with vehicle (non-allergic), or sensitized/exposed to OVA and treated with vehicle, $cTXA_2$, $cTXA_2$ + ISAP, or ISAP alone via minipump. A) flexiVent measurements of Elastance (E), Tissue Damping (G), Compliance (C), Newtonian Resistance (Rn), and Tissue Elastance (H) are shown after increasing doses of methacholine (MCH, 0 to 25 mg/ml). (B) Bar graphs of the airway parameters at the 25 mg/ml methacholine dose. N=15-20/group, *$p<0.05$ vs non-allergic mice (vehicle).

Supplemental Figure 7

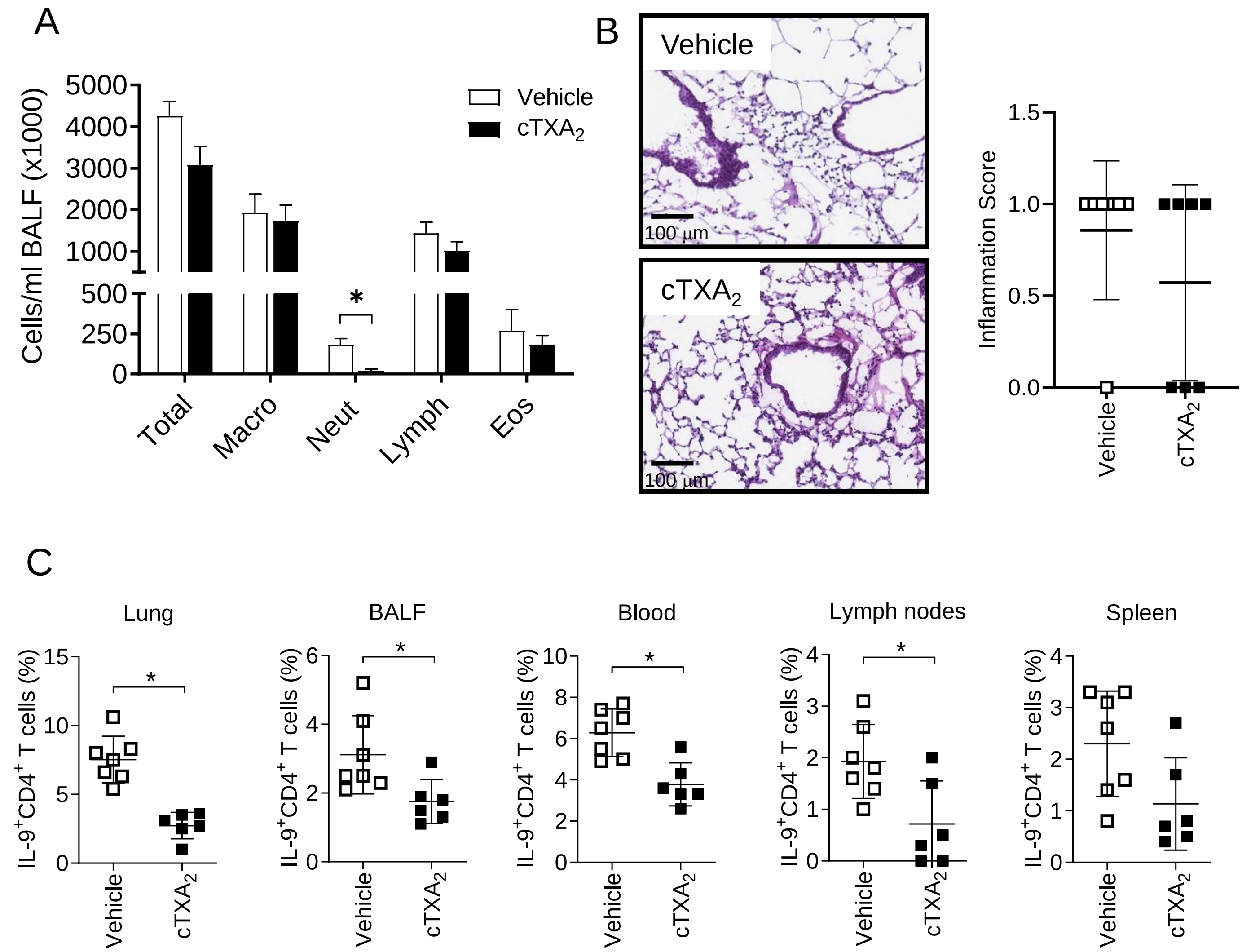

Supplemental Figure 7. $cTXA_2$ attenuates Th9 cells during allergic lung inflammation *in vivo.* Mice were sensitized with OVA/LPS and exposed to OVA via the airway in the presence of vehicle or $cTXA_2$ (delivered by osmotic minipumps) as described in Methods. (A) Total cell number and cell differentials from BALF were analyzed 48 hours after the last airway OVA exposure. Total neutrophil cell numbers were decreased in BALF from $cTXA_2$ -treated mice compared to vehicle-treated controls. (B) H&E-stained lung sections from vehicle- or $cTXA_2$-treated mice revealed no changes in overall inflammation or inflammation score. (C) $cTXA_2$-treated mice had a lower percentage of IL-9$^+$CD4$^+$ T cells in lung, BALF, blood and lymph nodes, but not spleen, than vehicle-treated mice. N=6-7 mice per group, *$p<0.05$.

Supplemental Figure 8

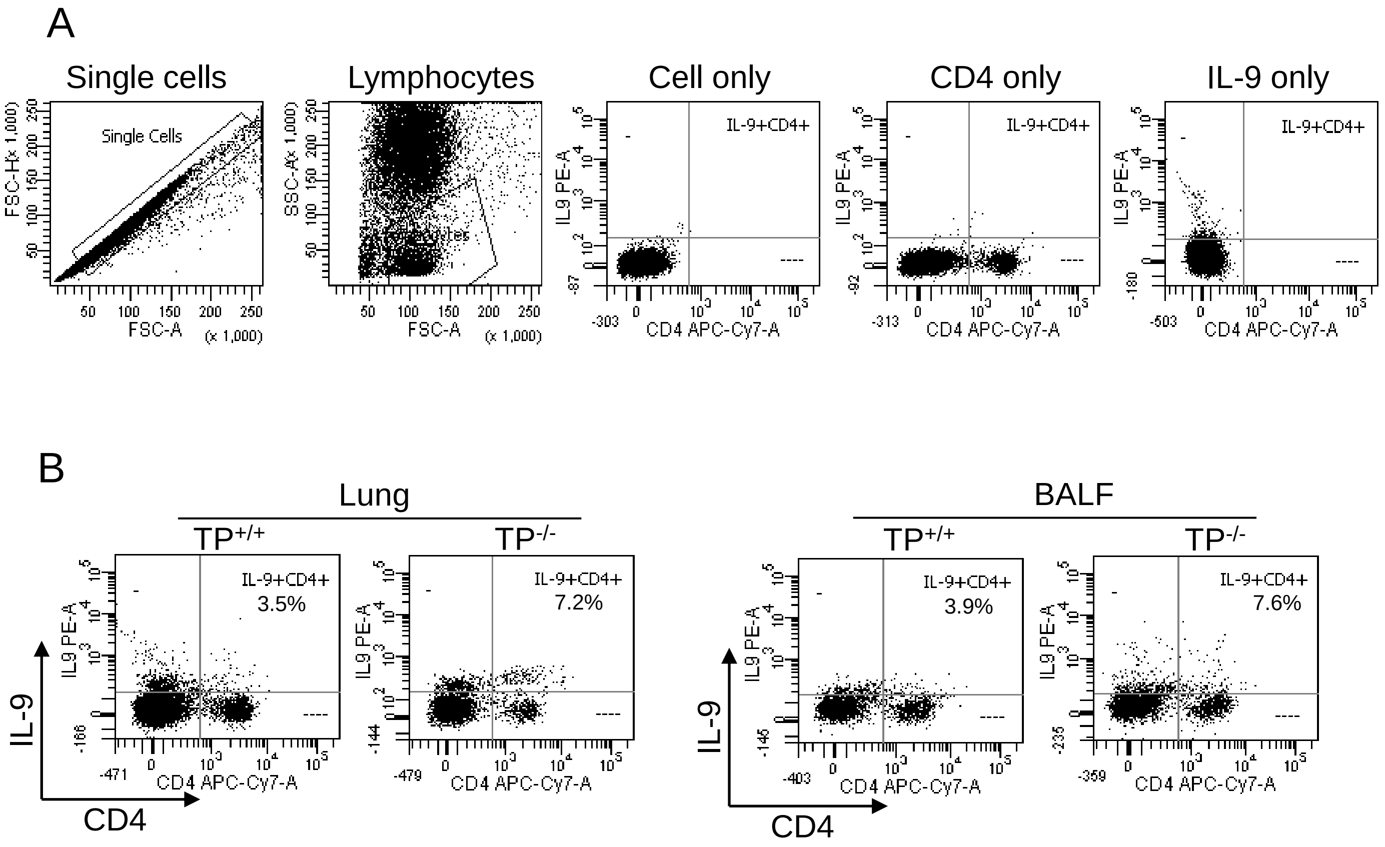

Supplemental Figure 8. A) Gating scheme for detection of IL-9$^+$CD4$^+$ T cells. Doublets and dead cells were excluded, and lymphocytes were electronically gated. IL-9$^+$CD4$^+$ T cells were identified as positive for both PE-conjugated anti-IL-9 and FITC-conjugated anti-CD4. B) Representative FACS dot plots for the data represented in Figure 2D. TP$^{+/+}$ or TP$^{-/-}$ mice were sensitized with OVA/alum and exposed OVA. IL-9$^+$CD4$^+$ T cells, as a percentage of CD4$^+$ cells in the lung and BALF after OVA-induced allergic lung inflammation (N=7-10 mice per group).

Supplemental Figure 9

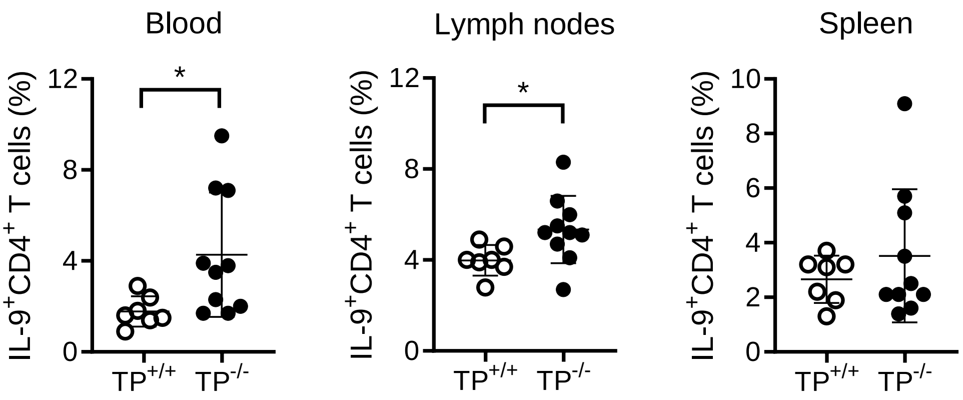

Supplemental Figure 9. TP receptor deficiency increases Th9 cells in blood and lymph nodes, but not spleen during allergic lung inflammation *in vivo*. $TP^{+/+}$ and $TP^{-/-}$ mice were sensitized with OVA/alum and exposed OVA via the airway as described in Methods. The percent IL-9^{+}CD4^{+} T cells in the blood, lymph nodes and spleen was measured by flow cytometry. N=7-10 mice per group, *p<0.05.

Supplemental Figure 10

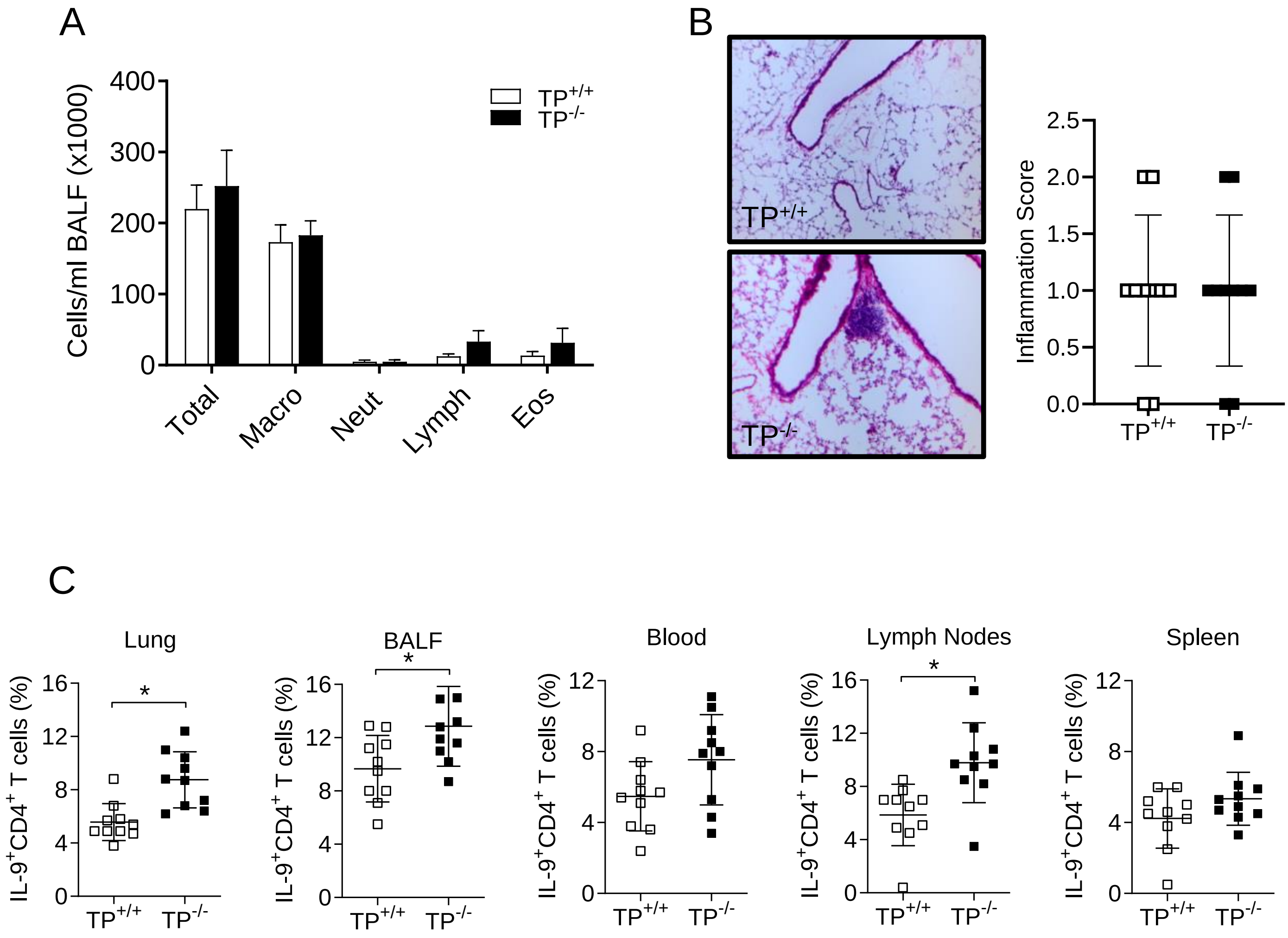

Supplemental Figure 10. Increased Th9 cell numbers in $TP^{-/-}$ mice during allergic lung inflammation *in vivo*. $TP^{+/+}$ and $TP^{-/-}$ mice were sensitized with OVA/LPS and exposed to OVA via the airway as described in Methods. (A) Total BALF cell number and cell differentials 48 hours after the last OVA exposure were similar in $TP^{-/-}$ and $TP^{+/+}$ mice. (B) H&E-stained lung sections from $TP^{+/+}$ and $TP^{-/-}$ mice revealed no changes in overall inflammation or inflammation score. (C) Lung, BALF and lymph nodes from $TP^{-/-}$ mice had a higher percentage of IL-9^+ $CD4^+$ T cells than respective tissues from $TP^{+/+}$ mice. N=10 mice per group, *$p<0.05$.

Supplemental Figure 11

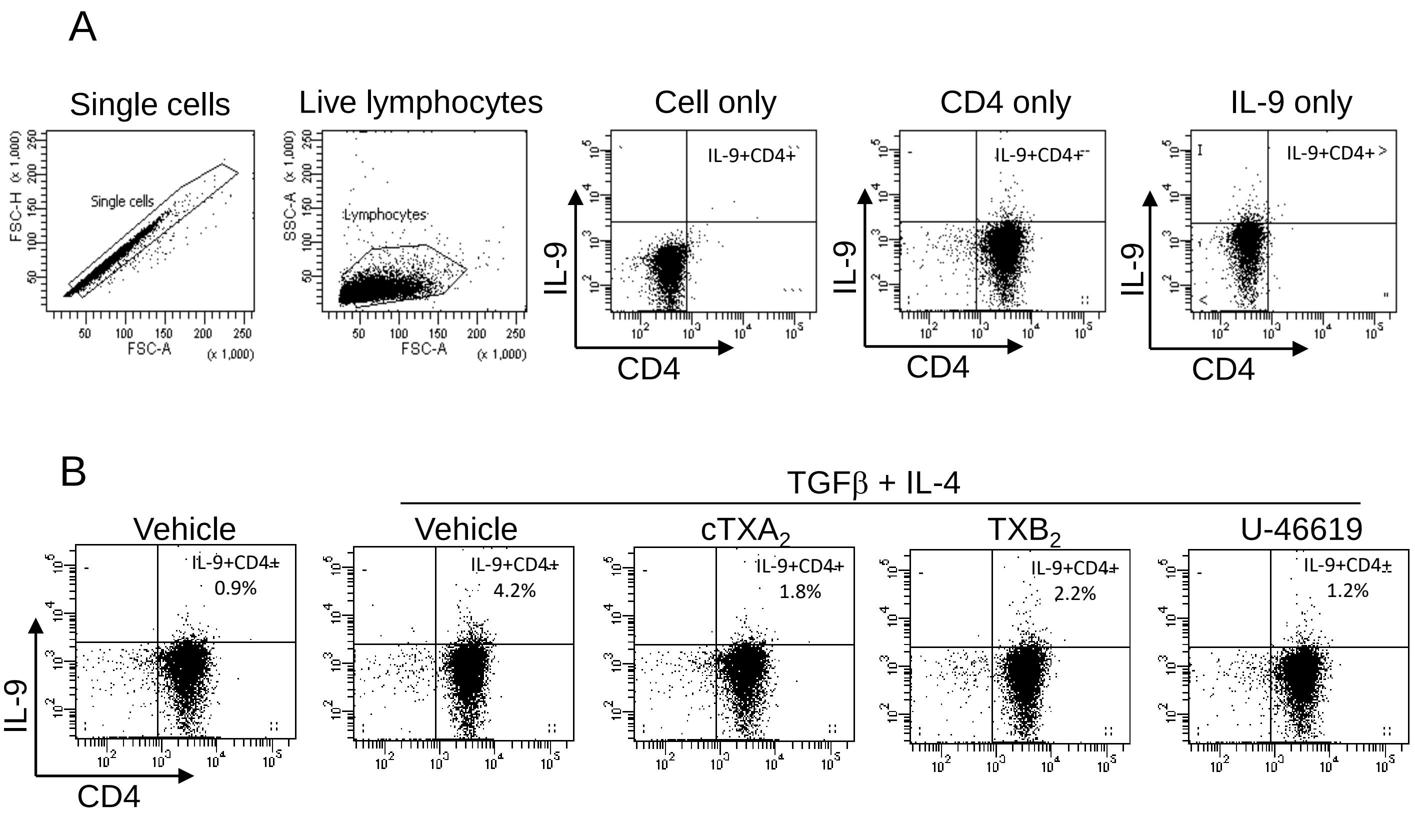

Supplemental Figure 11. $cTXA_2$ inhibits Th9 cell differentiation *in vitro*. Naïve $CD4^+$ $CD62L^+$ T cells were purified by FACS and treated with TGFβ + IL-4 to induce Th9 cell differentiation. A) Gating scheme for detection of IL-9^+CD4^+ T cells. Doublets and dead cells were excluded, and lymphocytes were electronically gated. IL-9^+CD4^+ T cells were identified as positive for both PE-conjugated anti-IL-9 and FITC-conjugated anti-CD4. B) Representative FACS dot plots for the data represented in Figure 3A. During Th9 cell differentiation, cells were treated with either vehicle, 300 nM $cTXA_2$, 500 nM TXB_2 (stable TXA_2 metabolite) or 500 nM U-46619 (TP agonists).

Supplemental Figure 12

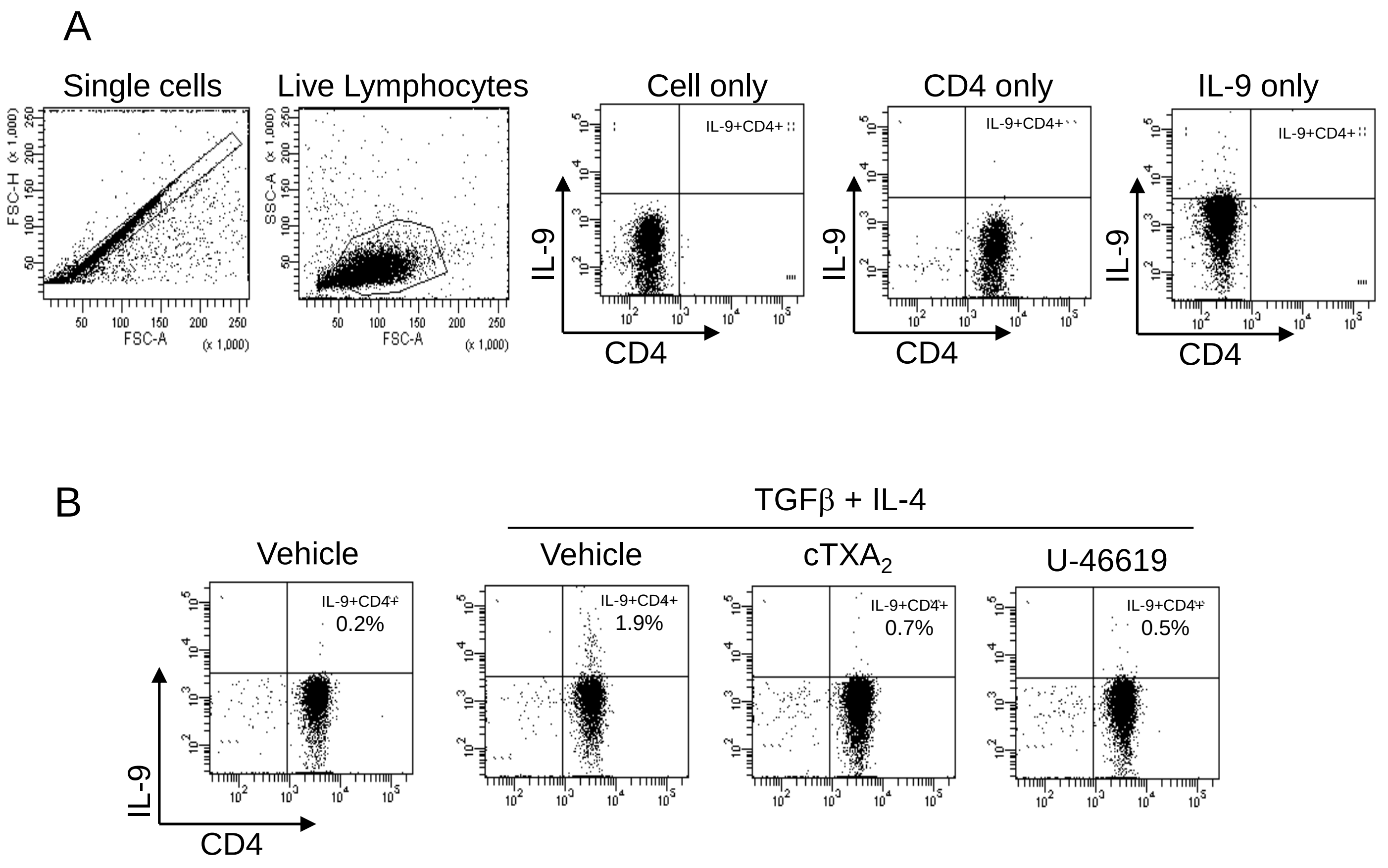

Supplemental Figure 12. $cTXA_2$ significantly inhibits human Th9 cell differentiation *in vitro*. Naïve $CD4^+CD62L^+$ T cells were purified from peripheral blood of healthy volunteers and treated with TGFβ + IL-4 to induce Th9 cell differentiation. During Th9 cell differentiation, cells were treated with either vehicle, 300 nM $cTXA_2$ or 500 nM U-46619. Representative FACS dot plot analyses are shown. B) Representative FACS dot plots for the data represented in Figure 4A. During Th9 cell differentiation, cells were treated with either vehicle, 300 nM $cTXA_2$, 500 nM TXB_2 (stable TXA_2 metabolite) or 500 nM U-46619 (TP agonists).

Supplemental Figure 13

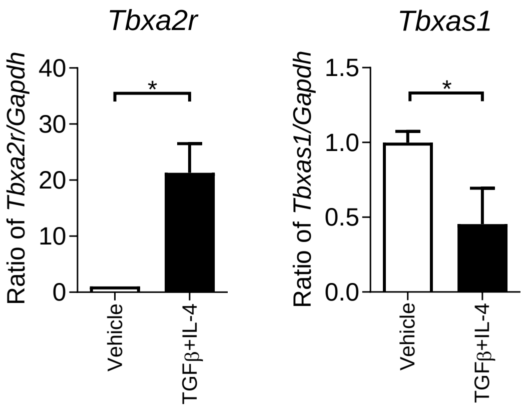

Supplemental Figure 13. $cTXA_2$ regulates *Tbxa2r* and *Tbxas1* mRNA levels during Th9 cell differentiation *in vitro*. Naïve $CD4^+$ T cells were purified by FACS and treated with vehicle or TGFβ + IL-4 in presence of anti-CD28 and anti-CD3 to induce Th9 cell differentiation *in vitro*. Compared to vehicle-treated controls, treatment with TGFβ + IL-4 significantly induced *Tbxa2r* mRNA and significantly repressed *Tbxas1* mRNA. N=4 per group, *p<0.05.

Supplemental Figure 14

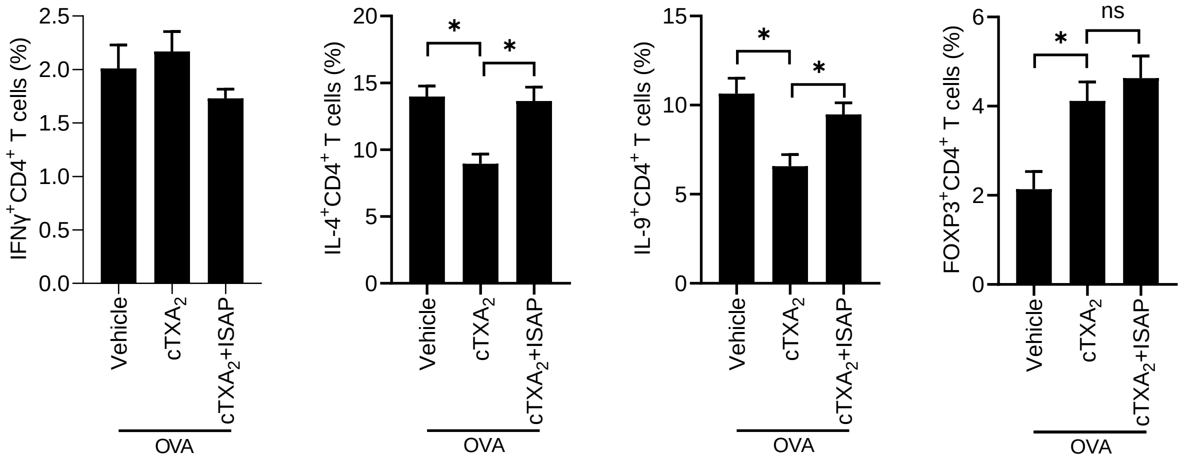

Supplemental Figure 14. Regulation of Th1, Th2, Th9, and Treg cells by $cTXA_2$ and ISAP in vivo. WT mice were sensitized/exposed to OVA and treated with either vehicle, $cTXA_2$ or TXA_2 + ISAP via minipump. Forty-eight hours after the last OVA exposure, mice were sacrificed and the percentages of lung Th1, Th2, Th9, and Treg cells were determined by FACS analysis. Neither $cTXA_2$ nor ISAP altered Th1 cell numbers as determined by the percentage of $IFN\gamma^{+}$ $CD4^{+}$ T cells. The significant reduction of Th2 ($IL4^{+}$ $CD4^{+}$) and Th9 ($IL9^{+}$ $CD4^{+}$) cell numbers by $cTXA_2$ was reversed by ISAP. $cTXA_2$ increased Treg cells ($FOXP3^{+}$ $CD4^{+}$); however, this effect was not attenuated by ISAP. N=10/group, $*p<0.05$.

Supplemental Figure 15

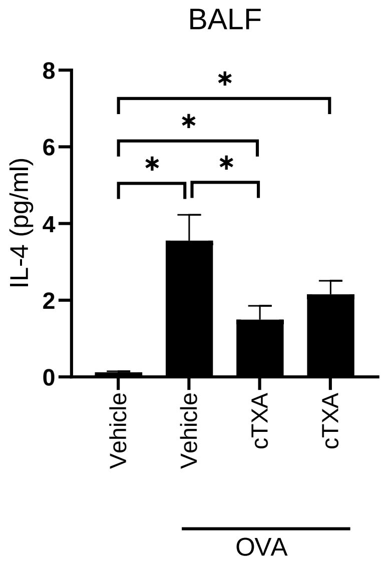

Supplemental Figure 15. Regulation of IL-4 levels in BALF from allergic mice treated with $cTXA_2$ with or without ISAP. WT mice were sensitized/exposed to OVA and treated with either vehicle, $cTXA_2$ or $cTXA_2$ plus ISAP via minipump. 48 hours after the last OVA exposure, mice were sacrificed and BALF IL-4 levels were determined by a Bioplex 200 using the IL-4 kit according to manufacturers instructions (Kit #171G5005M BioRad, Hercules, CA). N=9-14/group, *p<0.05.

Supplemental Figure 16

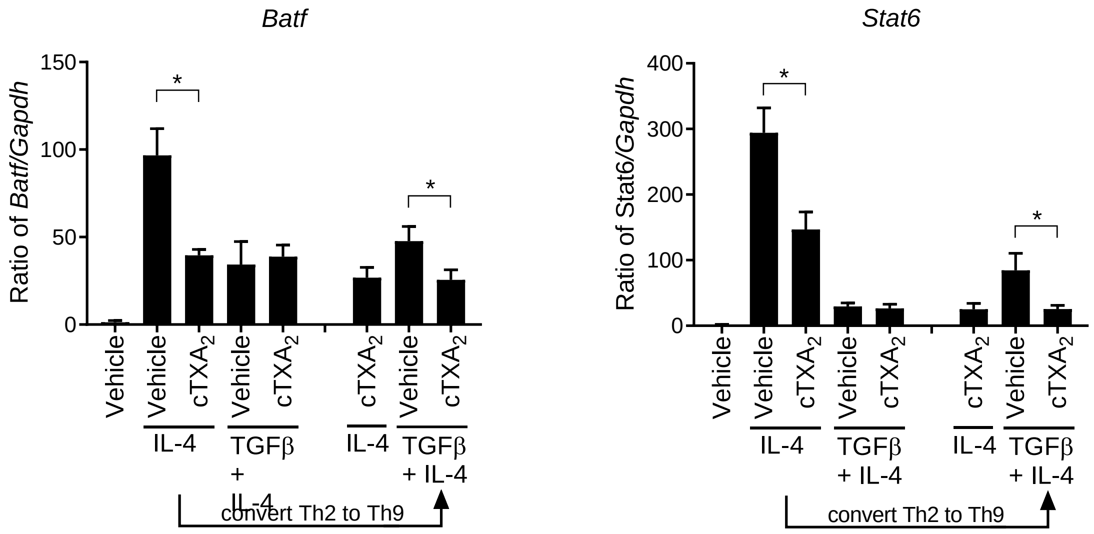

Supplemental Figure 16. $cTXA_2$ inhibits *Batf* and *Stat6* induction *in vitro*. Th9 cells can differentiate directly from naïve $CD4^+$ T cells or from Th2 cells *in vitro*. $cTXA_2$ strongly inhibits induction of *Batf* and *Stat6* mRNAs directly induced by IL-4, but not by TGFβ + IL-4. $cTXA_2$ also inhibits induction *Batf* and *Stat6* mRNAs in cells first differentiated to Th2 cells with IL-4 and subsequently differentiated to Th9 cells in the presence of TGFβ + IL-4. N=5, *p<0.05.

Supplemental Figure 17

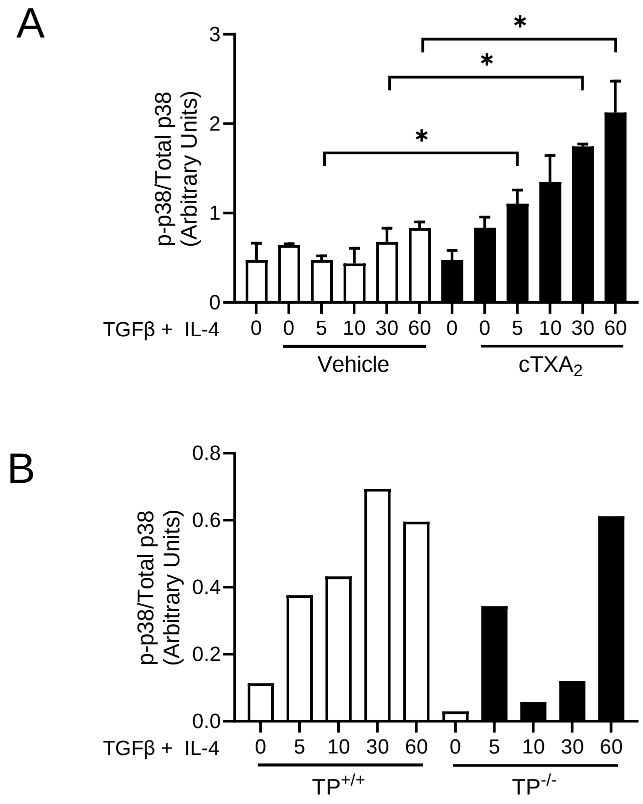

Supplemental Figure 17. Quantification of p38 phosphorylation in naïve T cells incubated with TGFβ and IL-4 and vehicle or $cTXA_2$. A) Quantification of phospho-p38/Total p38 staining in western blots of lysates from naïve T cells treated with TGFβ and IL-4 and vehicle or $cTXA_2$ as indicated here and depicted in Figure 8C. Data represent 3 independent experiments. N=3/group, *p<0.05. B) Quantification of phospho-p38/Total p38 staining in the western blots of lysates from naïve T cells from $TP^{+/+}$ or $TP^{-/-}$ mice treated with TGFβ and IL-4 indicated here and depicted in Figure 8C. N=1/group.

Supplemental Figure 18

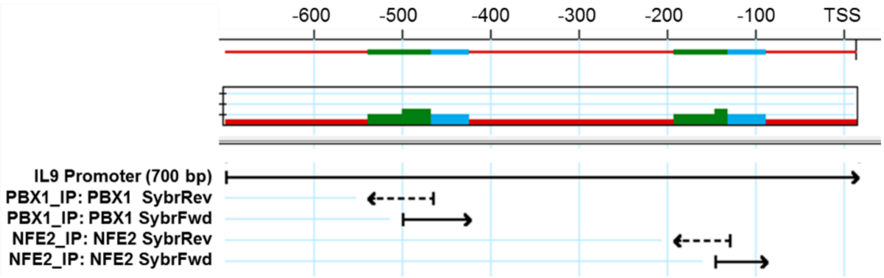

Supplemental Figure 18. ChIP-qPCR primers and PBX1 or NFE2 binding in the *Il9* promoter. The locations of ChIP-qPCR primers and PBX1 or NFE2 binding sites used in Figure 8 are shown relative to the *Il9* TSS (+1).

Supplemental Table 1

Plasmid Name	Proximal Promoter (bp)	chr 13 mm39 coordinates
IL9 v0 - 4.2 kb	-4202 to -1	56,630,060-56,634,261
IL9 v4 - 3.2 kb	-3210 to -1	56,630,060-56,633,269
IL9 v3 - 2.4 kb	-2449 to -1	56,630,060-56,632,508
IL9 v1 - 1.2 kb	-1249 to -1	56,630,060-56,631,308
IL9 v2 - 0.7 kb	-710 to -1	56,630,060-56,630,769

Supplemental Table 1. *Il9* proximal promoter fragments that were subcloned into the promoterless pGL4-10 luciferase reporter vector. Promoter fragments are listed relative to the transcription start site (TSS, bp -1) that is located on the negative strand of chromosome 13 at position 56,630,060.
