# Supplementary figures and images for: TXA2 attenuates allergic lung inflammation through regulation of Th2, Th9, and Treg differentiation
Source: J Clin Invest. 2024 Mar 14;134(9):e165689. doi: 10.1172/JCI165689 (PMC11060738; doi:10.1172/JCI165689)

Fig 7C Top

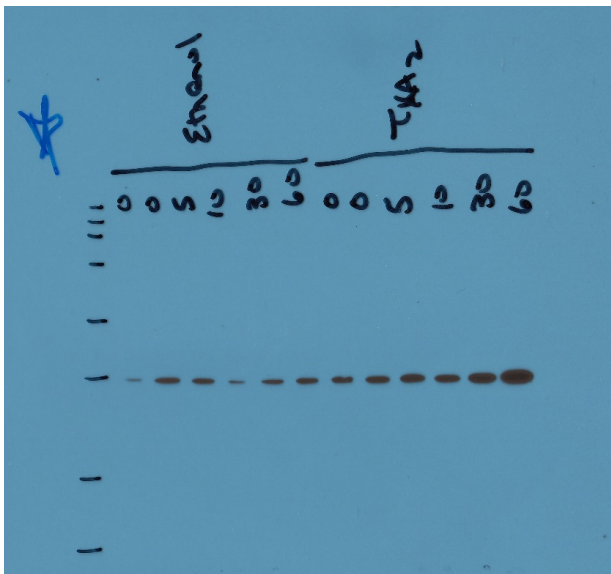

phospho-p38 (p-p38)

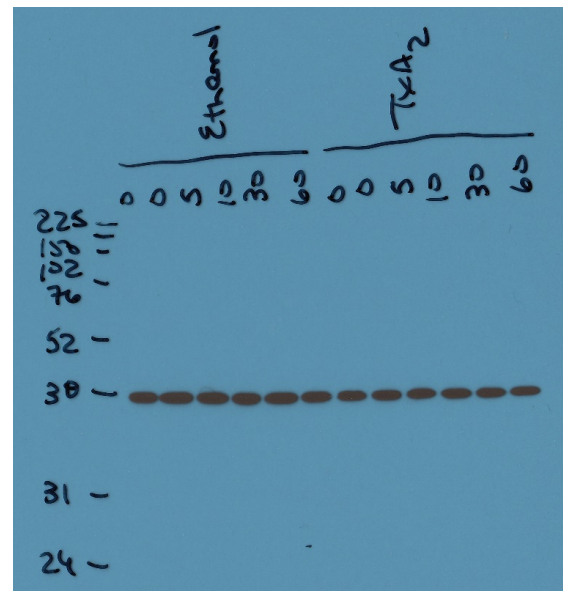

p38

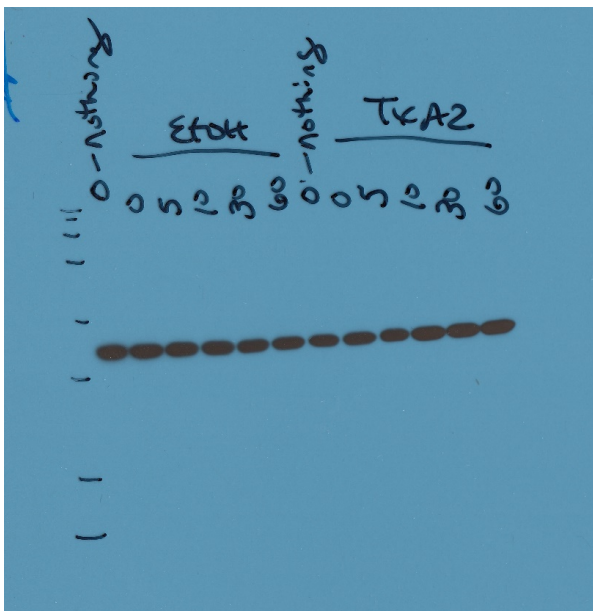

$\beta$ -actin

Fig 7C Bottom

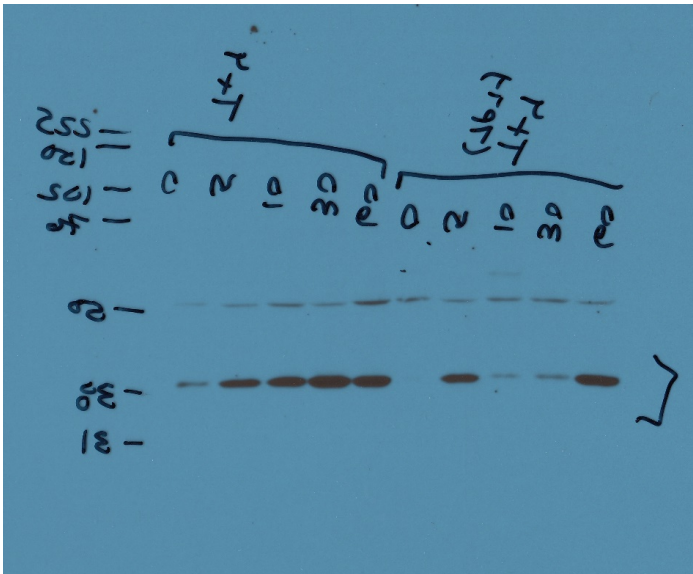

phospho-p38 (p-p38)

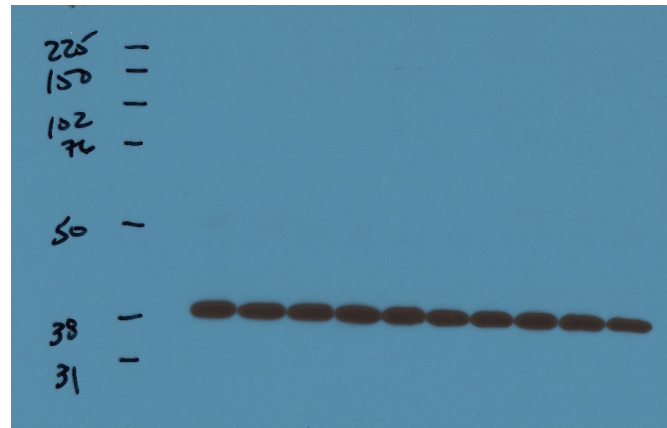

p38

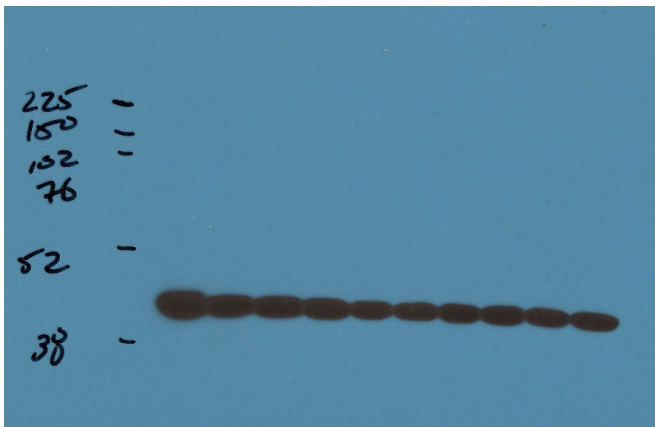

$\beta$ -actin

Supplement: Unedited blot and gel images [file jci-134-165689-s166.pdf]
